# Soundscape of green turtle foraging habitats in Fiji, South Pacific

**DOI:** 10.1371/journal.pone.0236628

**Published:** 2020-08-05

**Authors:** Elena Papale, Shritika Prakash, Shubha Singh, Aisake Batibasaga, Giuseppa Buscaino, Susanna Piovano

**Affiliations:** 1 BioacousticsLab, IAS Capo Granitola, National Research Council, Torretta Granitola, Italy; 2 Department of Life Science and Systems Biology, University of Torino, Torino, Italy; 3 School of Marine Studies, The University of the South Pacific, Suva, Fiji; 4 Ministry of Fisheries, Suva, Fiji; Department of Agriculture, Water and the Environment, AUSTRALIA

## Abstract

The soundscape features of the marine environment provide crucial information about ecosystem health for many species, and they are defined by the local biological, geophysical, and anthropogenic components. In this study, we investigated the soundscape at green turtle neritic foraging habitats in Fiji, South Pacific, with the aims of characterizing the contribution of each component and of comparing the levels of acoustic pressure among sites with different abundances of sea turtles. Four sites were selected at two islands, and one hydrophone was deployed at each site. Generalized additive models highlighted that sound pressure levels (SPLs) at low frequencies (125–250 Hz) were especially affected by wind conditions, while at higher frequencies (>250 Hz) SPLs were mostly influenced by fish and crustacean acoustic activity. Higher abundances of green turtles were found at sites with the highest levels of SPLs and the highest number of acoustic emissions by fishes and crustaceans but were not related to maximum seagrass and macroalgae coverage, or the highest number of fish. The selected coastal habitats have negligible anthropogenic noise, thus this study informs physiological and behavioral studies of the acoustic signatures that sea turtles might target and provides a baseline against which potential impact of soundscape changes on sea turtle spatial abundance and distribution can be evaluated.

## Introduction

The ecosystem soundscape can be considered a function of ecological variables, such as natural physical processes and meteorological events (geophonies), voluntary or involuntary biological sounds (biophonies), and noise generated by human activities (anthrophonies) [1]. Geophonies, biophonies, and anthrophonies are influenced by environmental and biological rhythms driven by diel, lunar, tidal, monthly, and seasonal cycles [2,3] and, as they interact and combine, they shape the temporal patterns and spectral composition of the soundscape [4,5]. Therefore, each ecosystem can be defined by a characteristic acoustic signature [6], determined by its unique combination of geophysical features, biological communities, and human interference [7–11], that is subject to temporal and spectral variability [4,12,13].

The contribution of sound components, as well as acoustic temporal variability, have been described for coral-dominated reefs [14–16], macroalgae-dominated reefs [6], and oyster reefs [17,18], in addition to sandy bottoms [6,19], estuarine habitats [13,20,21], maerl beds [10], coastal embayments [12,22], *Posidonia*-dominated seagrass meadows [19], and oceanic Arctic and Antarctic areas [11,23,24]. Together, acoustic temporal and spatial patterns reflect the ecological conditions of habitats, as well as the acoustic adaptation of soniferous species to the environment [25–27]. As a result, many marine vertebrates and invertebrates, at multiple life-history stages, rely on an integrated sensory response incorporating acoustic and other stimuli for orientation during navigation and selection of suitable habitat [16, 28–30 but see also 31]. However, different sets of stimuli are likely used at different spatial scales [29]. For example, visual and mechanical cues are reliable over short (from centimeters to tens of meters) distances [32,33] while, depending on their intensity, acoustic cues can support habitat selection at short or long spatial scales [29,34,35].

Acoustic cues and their spatial and temporal variability likely provide marine animals with an ‘acoustic image’ of the underwater habitat structure and its community composition [36]. The underlying assumption, known as the ‘soundscape paradigm’, is that animals can perceive and interpret the acoustic stimuli [1,4,37]. However, little is known about the cognitive capacities of many marine species [37]. For example, the use of acoustic cues in habitat selection has been largely investigated in fishes and mammals that produce sounds; however, species-specific audiograms are available for <1% of marine fishes and ca. 25% of the marine mammals (see review in [38]), resulting in uncertainty regarding specific hearing thresholds.

In the marine environment, acoustic cues can be represented by the sound produced from high-energy waters, such as tidal currents, breaking waves, and wind, which commonly extend below 2 kHz [39], as well as by biological signals such as those generated by vertebrates and invertebrates. Fish signals, for example, are made of pulsations often below 1 kHz [40] and show high diel and seasonal variability [12]. In seagrass meadows and coral reef habitats, the loudest biological contribution that dominates the soundscape is provided by invertebrates, mainly crustaceans such as snapping shrimps (genera *Alpheus* and *Synalpheus*) [18,41–44]. In tropical marine habitats, their short broadband pulses (<0.1 s, in [45]) can drown out all the other components at frequencies over 2 kHz [46] and contributes to habitat selection by pelagic larvae of corals, fishes and crustaceans [14,30,35,47,48]. Different acoustic communities create spatial variability. For example, at coral reefs in Hawaii, the frequencies of sounds produced by fishes range from <100 Hz to 1 kHz during non-feeding behavior (with peaks at 100–300 Hz), to 2–6 kHz during feeding behavior [49]. In Australia, they range from 100 Hz to 2 kHz [50], while in Panama and Florida they dominate the 400 Hz and 25–200 Hz bands, respectively [2].

Similarly, sea turtles likely use acoustic stimuli together with a variety of other potential cues such as magnetism, light, colors, and water chemicals to orient over long and short spatial scales during their different life stages, i.e., post-hatchling, pelagic juvenile, neritic juvenile and adult [51–54]. The life cycle of hard-shell sea turtles is characterized by a terrestrial and a marine phase. In general, the terrestrial phase is relatively short, accounting for egg incubation in sandy beaches, hatchling emergence from the egg chamber, and subsequent crawling activity to reach the sea. In addition, mature females cyclically return to their natal beach to nest. The marine phase accounts for most of the sea turtles’ lives and is characterized by complex migration patterns that include periodic returns to specific geographic areas, such as coastal foraging grounds [52]. After an initial oceanic stage, sea turtles settle in a neritic environment and often stay in small home ranges for several years [55] and select, at a finer scale, specific resources available within neritic habitats [56–59]. However, sea turtles in the neritic stage can travel across oceanic waters when moving between different foraging grounds, and adult individuals migrate across oceanic waters to reach (and return from) their mating and nesting areas.

Hard-shell sea turtles are capable of detecting and responding to acoustic stimuli within a frequency range of 50 Hz to 2 kHz in both aerial and seawater media (Table 1). The green turtle (*Chelonia mydas*) and the loggerhead turtle (*Caretta caretta*) have been the focus of the majority of the acoustic-detection studies. The green turtle reportedly has the broadest underwater hearing range, from 50 Hz (95 dB re: 1 μPa-rms) to 1.6 kHz (157 dB re: 1 μPa-rms) [54]. The loggerhead turtle appears to have a narrower underwater hearing range, from 50 Hz (101 dB re: 1 μPa-rms) [60] to 1.13 kHz (148 dB re: 1 μPa-rms) [61]. The loggerhead turtle is also the only sea turtle species in which hearing capabilities have been investigated at different life stages. Studies show consistency in the frequencies detected from hatchlings to juveniles and adults loggerhead turtle, despite the different environments where they live. Lavender et al. [60] suggested that loggerhead turtles have a basic hearing sense or, alternatively, hear low frequencies, which is useful in the coastal environment where hatchlings are born and neritic-stage animals (e.g. large juveniles and adults) live, and is simply maintained during the initial oceanic stage and the migration performed by large juveniles and adults.

**Table 1:** Hearing capabilities in sea turtles, by species. Unless otherwise stated, the intensity is expressed as dB re: 1 μPa.

Species	Medium of the emitted stimulus	Life stage	Hearing range, in Hz (dB)	Highest sensitivity, in Hz (dB)	Procedure*	Reference
*Caretta*	underwater	post-hatchling	50 (124)– 1,100 (134)	200 (116)	Auditory evoked potentials	[60]
	underwater	post-hatchling	50 (101)– 800 (116)	200 (85)	Behavioral	[60]
	underwater	juvenile	50 (117)– 1,100 (140)	50 (117), 100 (118), and 400 (118)	Auditory evoked potentials	[60]
	underwater	juvenile	50 (103)– 1,000 (102)	800 (76)	Behavioral	[60]
	underwater	Adult	100 (112)– 1,131 (141)	200 (110) and 400 (110)	Auditory evoked potentials	[61]
	underwater	Adult	50 (110)– 800 (148)	100 (98)	Behavioral	[61]
	Aerial	post-hatchling	100 (92)– 900 (94)	500 (81) and 600 (84)	Auditory evoked potentials	[62]
	aerial	juvenile	100 (97)– 800 (122)	600 (94) and 700 (96)	Auditory evoked potentials	[63]
	Aerial	juvenile	100 (87)– 700 (98)	100 (87), 400 (87), and 500 (86)	Auditory evoked potentials	[62]
	vibrational	juvenile	250 (-30 dB re: 1g rms)– 1,000 (-10 dB re: 1g rms)	250 (-30 dB re: 1g rms)	Auditory evoked potentials	[64]
*Chelonia mydas*	underwater	juvenile	50 (95)– 1,600 (157)	200 (87), 300 (85), and 400 (88)	Auditory evoked potentials	[54]
	Aerial	juvenile	50 (18 dB re: 1 dyne/cm^2^)– 2,000 (39 dB re: 1 dyne/cm^2^)	400 (-35 dB re: 1 dyne/cm^2^)	Cochlear response potentials	[65]
	Aerial	juvenile	100 (101)– 800 (119)	600 (94) and 700 (96)	Auditory evoked potentials	[62]
	Aerial	juvenile	50 (80 dB re: 20 μPa-rms)– 800 (78 dB re: 20 μPa-rms)	400 (44 dB re: 20 μPa-rms)	Auditory evoked potentials	[54]
	aerial	subadult	100 (93)– 500 (108)	300 (83)	Auditory evoked potentials	[63]
	Aerial	subadult	100 (96)– 500 (106)	200 (93) and 400 (91)	Auditory evoked potentials	[62]
	vibrational	juvenile	30 (18 dB re: 1 dyne/cm^2^)– 700 (21 dB re: 1 dyne/cm^2^)	300 (-11 dB re: 1 dyne/cm^2^) and 500 (-12 dB re: 1 dyne/cm^2^)	Cochlear response potentials	[65]
*Lepidochelys kempii*	Aerial	juvenile	100 (110)– 500 (113)	100 (110), 200 (110). and 500 (113)	Auditory evoked potentials	[62]
	Aerial	juvenile	100 (104)– 500 (115)	100 (104) and 200 (106)	Auditory evoked potentials	[63]

* Auditory evoked potentials are electric responses generated by the brain in response to the acoustic stimulation of the nervous system, whereby electrodes are used to detect voltages.

Both green and loggerhead turtles respond to underwater acoustic stimuli that overlap in frequency with fish-generated sounds and with common geophonies. In addition, both sea turtle species are likely capable of perceiving invertebrate-generated sounds at low frequencies: although this ability greatly depends on the sound source level, the active space of the sound, and the distance between the source and the receiver. For the same reasons, both sea turtle species likely miss out on the highest frequencies reached by the snapping shrimps [66]. The suggested ecological advantage of being able to detect low-frequency sounds, which travel farthest in the marine environment when produced at high intensities and dominate the soundscape [67], is associated with the possible use of acoustic stimuli as environmental cues [54]. Furthermore, recent studies proved the social role of vocalizations in freshwater turtles. For example, the giant South American river turtle (*Podocnemis expansa*) uses acoustic communication during the nesting season to facilitate synchronization of group migration to nesting beaches [68], as well as post-hatching parental care [69], while the northern snake-necked turtle (*Chelodina oblonga*) uses acoustic communication during the breeding season as a display to attract a mate in water with poor visibility [70].

This study aims to: 1) investigate the relationship between green turtle abundance and habitat quality data at tropical green turtle neritic foraging habitats, 2) characterize the levels of acoustic pressure present at each site, 3) assess the contribution of the acoustic components to the soundscape, and 4) infer the relationship between local soundscapes and green turtle abundance. Characterization of the acoustic stimuli present in a sea turtle coastal habitat with negligible anthropogenic noise has a twofold advantage. First, it will provide a better understanding of the biological significance of sea turtle hearing, as it could inform physiological and behavioral studies of the acoustic signatures that sea turtles might target [60]. Second, it will form a baseline against which changes in habitat soundscape (potentially associated with altered ecological communities in response to climate change or anthropogenic acoustic pollution) and their impact on turtle distribution can be evaluated.

## Materials and methods

### Ethics statement

All sea turtle handling procedures were approved by the Animal Ethics Committee of The University of the South Pacific and performed under a Ministry of Fisheries Exemption Permit (Ref. FI/G/12-5).

### Study area

**Fig 1:**
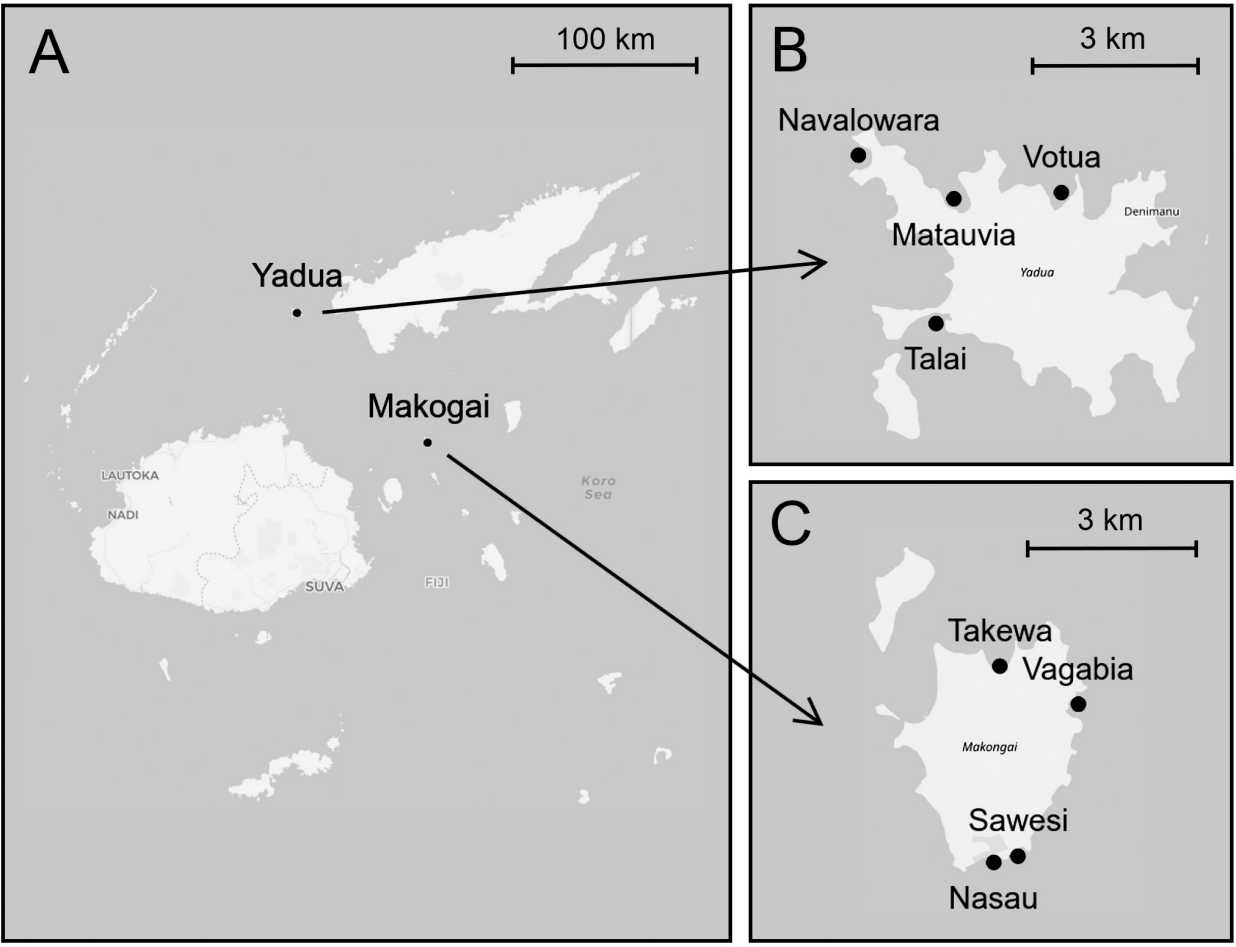
Study area. (A) Main islands of Fiji (South Pacific Ocean) and relative position of Yadua Island and Makogai Island. (B) The four sites of hydrophone deployment at Yadua Island. (C) The four sites of hydrophone deployment at Makogai Island. Image prepared in R [73] with package “leaflet” [74], geodata from OpenStreetMap (data are available under the Open Database License 1.0, cartography is licensed under the Creative Commons Attribution-ShareAlike 2.0 license). Image retouched with GIMP [75].

Fieldwork was carried out in 2018 at Makogai Island and Yadua Island in Fiji (Fig 1A), tropical South Pacific, where juvenile green turtles from different stocks form foraging aggregations in shallow coastal waters [71,72]. The surface areas of Makogai and Yadua Islands are about 8 km^2^ and 15 km^2^, respectively. Several back-reef embayments (hereafter “sites”) are present in both islands and used by green turtles for feeding. Based on previous data on green turtle distributions, green turtles move among sites within the same island, but not between islands (S. Piovano unpublished data). This study focused on four sites on each island (Table 2, Fig 1B and 1C). The sites ranged in area from 14,600 m^2^ to 68,300 m^2^, and consisted of neritic habitats extending from the intertidal zone (semidiurnal tidal cycle) to the subtidal zone (max depth: mean ± SD = 5.0 ± 1.9 m). All sites are delimited by coral reefs on the oceanic side and are mostly fringed by sandy beaches on the terrestrial side.

**Table 2:** Description of the eight green turtle foraging sites at Makogai Island and Yadua Island, Fiji, South Pacific.

Site	Dominant landside environment	Max depth (m)	Surface (m^2^)
Makogai Island			
Nasau	sandy beach	3	28,800
Sawesi	sandy beach	5	28,000
Takewa	sandy beach	4	14,600
Vagabia	mangrove vegetation	3	64,600
Yadua Island			
Matauvia	sandy beach	6	45,400
Navolawara	sandy beach	8	30,900
Talai	sandy beach	4	47,500
Votua	mangrove vegetation	7	68,300

### Data collection

Local soundscape and abundances of green turtles were recorded at each of the eight sites during the same periods: from 19 to 22 September 2018 at Makogai Island, and from 30 September to 3 October 2018 at Yadua Island. To minimize interference, fish counts and benthic surveys for habitat characterization were performed after completion of the sound recording period.

### Acoustic data

Four underwater autonomous acoustic recorders (hereafter “UAARs”) were deployed at each island in the ratio of one UAAR per site. Deployment and collection of UAARs were done from a small boat at high tide. Each instrument was synchronized before deployment to simultaneously record uninterrupted sequences of 2-min files for four consecutive days. Unfortunately, one UAAR malfunctioned and recorded for less than 24 hours. An average 64.7 hr per site (SD = 28.2 hr per site) was recorded, at a sample rate of 48 kHz at 16 bits. The UAARs were located 1 m from the soft sea bottom, anchored with a 30 kg weight at a depth ranging from 3 m to 8 m, and kept vertical by a submerged buoy (S1 Fig). The instruments deployed were omnidirectional calibrated hydrophones with a flat sensitivity response of -174.5 (± 2) dB re: V/μPa from 100 Hz to 100 kHz (Benthowave Low Noise Broadband Hydrophone with Preamplifier, model BII-7016 T6) and a digital signal processor (Texas Instruments, model TMS320C5535), coupled with a stereo audio codec (Texas Instruments, model AIC3204).

### Habitat characterization and sea turtle abundance

Fish counts were performed at each site by counting the number of fishes (by species or by genus, whatever possible) visible in underwater videos recorded with pocket cameras (GoPro Hero 4) along three belt transects at high tide. Each transect had a fixed width of 5 m (whose sides and middle lines were marked with measuring tapes) and an average length of 89 m (SD = 16.4 m) resulting from a maximum length of 100 m or until reaching the coral reef crest, whichever was encountered first. Fish count data were standardized as the number of fishes per 10 m, counted as an average of two swims. Each video lasted an average of 26.6 min (SD = 13.0 min), depending on transect length. To allow disturbed fishes to resume their normal activity, the two underwater operators undertook five minutes of no activity after laying the transect lines. Quadrat surveys to quantify habitat characteristics [76] were conducted after underwater video recording for fish counts. The biodiversity, distribution and percent coverage of seagrasses and macroalgae at each site were recorded within standard 100 × 100 cm quadrats placed every 10 m along the midline of the same belt transects used for the fish count. Quadrats were also used to assess substrate composition. Tide heights were estimated from the Fiji Meteorological Service recording at Levuka station (17° 40’ 00” S, 178° 48’ 00” E) by using the software WXTide32 4.6. Wind intensity and direction were obtained from Fiji Meteorological Service at Ratu Kadavulevu School Lodoni station (17° 44’ 27” S, 178° 33’ 04” E) for Makogai Island, and Seaqaqa station (16° 32’ 23” S, 179° 8’ 23” E) for Yadua Island.

The abundance of sea turtles at each site was recorded as the sum of sighted and captured sea turtles during a total of 16 hours of surveys performed during the days of UAARs deployment. Each site was monitored for 2 hr (30 min × 4 days) by boat at high tide. The capture of sea turtles was performed via “rodeo”, a standard technique that consists of the hand-capture of sea turtles by diving onto them out of a small boat [77–80]. This method might have caused some disturbance, but it was the best possible approach. Before being released back into the water, each turtle was tagged individually with an Inconel flipper tag (National Band and Tag Company, Style 681) attached to the trailing edge of the right front flipper [81], and species and minimum curved carapace length were recorded. In addition, during each survey, all turtles sighted but not captured were noted. The relative abundance of sea turtles observed during this study was used to categorize each site as low abundance (<2 turtles/10,000 m^2^), moderate abundance (2–3.9 turtles/10,000 m^2^), and high abundance (≥4 turtles/10,000 m^2^).

### Acoustic analyses

Acoustic analyses were conducted on each 2-min file, except for those recorded during turtle rodeos. To describe the general acoustic pressure for each site, sound pressure level values (SPL dB re: 1 μPa-rms, hereafter “SPL”) were measured through SPL analysis at 1/3 octave bands, centered from 16 Hz to 16 kHz [82] using the software Avisoft-SASLab Pro, calibrated to provide absolute dB values. SPL values at each frequency band were transformed from dB to Pa for calculation of mean and standard deviation, which are reported after back-transforming Pa into dB.

The dial cycle of the SPLs at the 1/3 octave bands, centered at 31.5 Hz, 125 Hz, 250 Hz, 500 Hz, 1000 Hz, and 2000 Hz, was examined to study temporal rhythms in sound levels and the daily abundance of the environmental and biological variables in the soundscape. Selected bands had a low correlation among them.

Twelve small boats, less than 10 m in length, are present at the islands (ten at Yadua Island and two at Makogai Island, including the one used during this research). The boats are equipped with outboard engines, and used to go fishing around the island and, when needed, to travel from the village to mainland (and back). To assess anthrophonies, the passages of the motorboats were counted through visual and aural inspection of the spectrograms.

The contribution of geophonies was evaluated using tide height and wind intensity as a proxy for high-energy waters [9,22]. Tide height and wind intensity were compared to SPL values at the different bands to infer their influence on background noise intensity.

**Fig 2:**
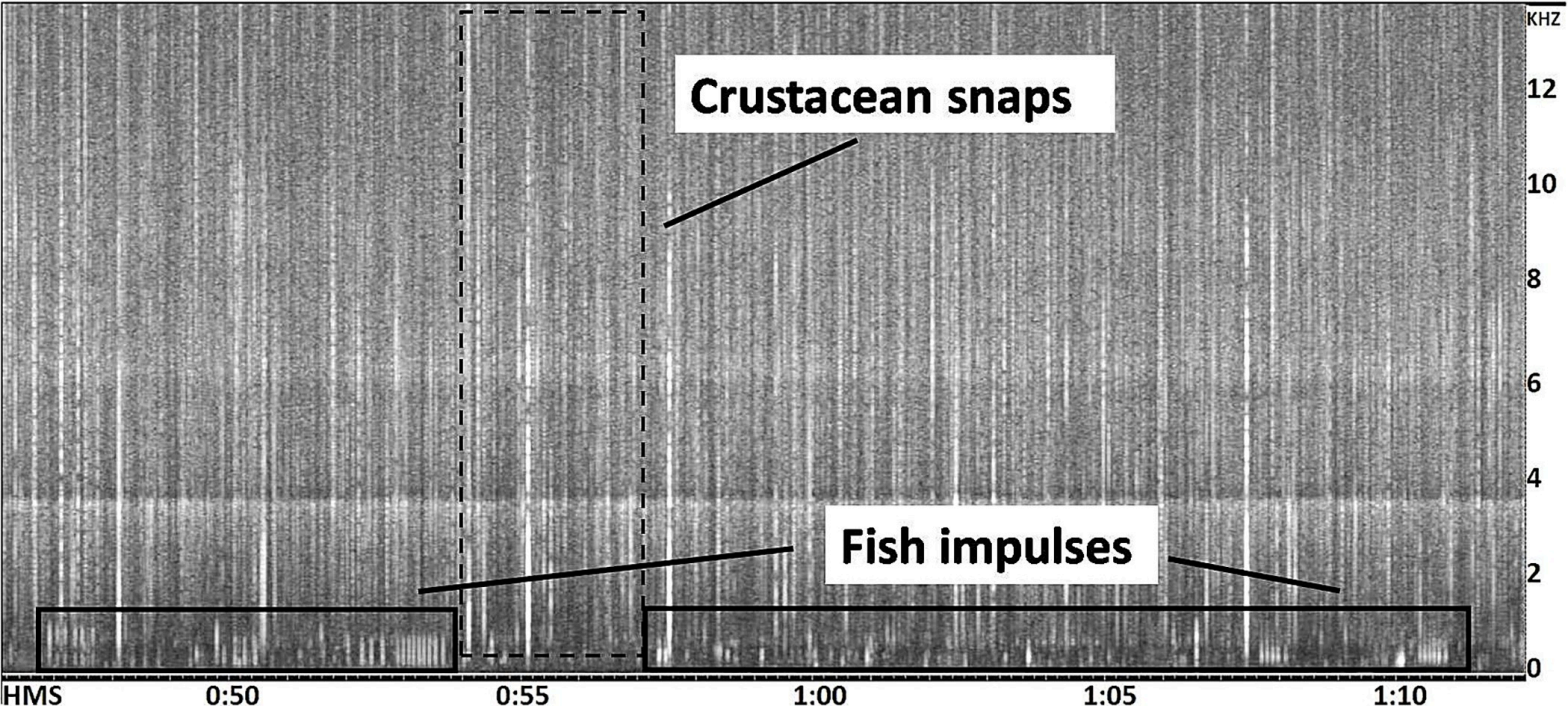
Spectrogram (frequency vs time) of a portion of a recording at Vagabia at 18:00. Squares highlight details of crustacean snaps (dashed line) and, at a lower frequency, fish impulsive signals (continuous line).

The contribution of biophonies was evaluated using the number of acoustic events attributed to fishes and crustaceans as biological indicators of acoustic activity. Sounds of fishes and snapping shrimps were detected automatically with a custom-made code in Matlab^TM^ 2019, irrespective of the different types of sounds produced by the different species (only the band pass filter was changed between fishes and snapping shrimp). Most fish sounds consisted of pulses at frequencies between 100 Hz and 800 Hz (see Fig 2), so, each 2-min file was down-sampled at 2 kHz and then filtered with a band pass at 100–800 Hz (equiripple, order 20). To count shrimp snaps in each 2-min file, the code filtered the signal (without down-sampling) with a band pass filter at 4–9 kHz (equiripple, order 20). This band pass filter was used to improve the identification of snapping shrimp signals by excluding low-frequency pulses produced by fishes and by photosynthetic activity [83]. Then, the code applied the Teager Kaiser filter and counted all the pulses that overcame an empirical threshold [84,85], one for fish and another one for snapping shrimps; these two thresholds were the same for all of the eight sites. Before applying the code to all the files, the accuracy of the detection was validated by manually counting the number of clicks from a randomly selected subsample (5%) of the audio files, and resulted in 95% correct identification of impulsive signals, i.e., fish pulses and snapping shrimp snaps.

### Statistical analysis

Statistical differences in curved carapace length of captured green turtles at the two islands were explored with a Wilcoxon-Mann-Whitney test. The correlation among SPL values at the frequency bands selected wind conditions, tide height, and biological acoustic signals were assessed using a Spearman correlation test (r_s_). Furthermore, since frequency bands from 100 Hz could be influenced by several variables, we tested which predictors better explained the variation of SPL values by using a generalized additive model (“GAM”) (R “mgcv” package version 1.8–2; [86]). SPL values were chosen as the response variable and tested as a function of the predictors *hours* (both daytime and nighttime, discrete variable ranging from 0 to 23), *wind condition* (mean Beaufort wind condition for each 2-min file), *fish* (number of fish acoustic emissions recorded in each 2-min file) and *crustaceans* (number of snapping shrimp clicks recorded in each 2-min file). A Gaussian distribution and identity link function were chosen. A penalized cubic regression spline was selected for the explanatory variables, except for *hours*, for which a cyclic cubic regression spline was used. SPLs at the frequency bands considered were compared among sites by using a Kruskal-Wallis test. Sites with less than 24 hours of recording were excluded from circadian and biological analyses. All analyses were performed in R [87].

## Results

### Habitat characterization and sea turtle abundance

The eight sites presented several differences (Table 3). The dominant substrate was often sand, but mixed substrates and rock substrates were also recorded. In general, seagrass coverage was primarily composed of *Halodule pinifolia*, *H*. *uninervis*, *Halophila ovalis*, and *Syringodium isoetifolium*, with a prevalence of *H*. *pinifolia* at Yadua Island (58% of seagrass coverage), and *H*. *uninervis* (71% of seagrass coverage) at Makogai Island. The average number of fishes counted every 10 m ranged from 2.0 to 15.8. A total of 83 sea turtles were observed, of which 75% were sighted and 25% were captured. All were green turtles, except for two hawksbills (*Eretmochelys imbricata*) that were captured at Yadua Island and excluded from the statistical analysis. Based on green turtle relative abundance during the study period, two sites were ranked as high abundance, three as moderate abundance, and three as low abundance (Table 3). The two sites with a higher relative abundance of green turtles (i.e., Nasau and Vagabia) had a moderate to low average coverage of seagrass, moderate to high coverage of macroalgae, and a moderate to high number of fish observed, compared to the other sites. The lower relative abundance of green turtles was recorded at three sites (Matauvia, Navalowara, and Takewa) that had null or high average coverages of seagrasses, low to moderate coverages of macroalgae, and a moderate to high number of fish observed compared to the other sites. There was no statistically significant difference in green turtle size between the two islands (curved carapace length: median = 53 cm, mean ± SD = 54.5 ± 8.1 cm at Makogai Island and median = 53 cm, mean ± SD = 55.9 ± 6.9 cm at Yadua Island; Z = -0.60, p = 0.567), confirming previous findings by Piovano et al. [59] in 2015–2016.

**Table 3:** Habitat characterization of each green turtle foraging site at Makogai Island and Yadua Island, Fiji, South Pacific.

Site category	Site	Dominant substrate (mean %)	Seagrass % coverage (mean ± SD)	Macroalgae % coverage (mean ± SD)	Number of fish/10 m	Number of green turtles/10,000 m^2^
high	Nasau	sand (78)	3.4 ± 4.3	46.7 ± 35.9	15.8	9.8
high	Vagabia	sand (83)	9.4 ± 13.8	10.3 ± 19.8	7.4	4.8
moderate	Sawesi	mixed (rock 39, rubble 36)	1.2 ± 1.2	39.3 ± 28.7	2.0	3.2
moderate	Talai	sand (55)	34.1 ± 40.8	21.4 ± 25.7	4.9	2.0
moderate	Votua	sand (71)	31.5 ± 4.4	15.1 ± 15.5	5.7	2.0
low	Matauvia	sand (100)	38.7 ± 20.6	0.7 ±3.7	6.1	1.4
low	Navolawara	mixed (sand 26, dead corals 24, rubble 22)	-	12.7 ± 19.4	12.7	1.0
low	Takewa	rock (45)	-	7.7 ± 19.1	6.1	1.0

Wind direction (S2 Fig) concentrated around 145° (i.e., S-SE) and 245° (i.e., W-SW) at Makogai Island, whereas it converged prevalently from 100° to 155° (i.e., SE) and rarely around 360° (i.e., N) at Yadua Island. Wind intensity consistently showed higher levels at Yadua Island (mean Beaufort level ± SD = 4.07 ± 1.02) compared to Makogai Island, where a larger range of wind intensity was recorded (mean Beaufort level ± SD = 1.89 = ± 1.10). A strong peak was recorded during daylight hours at both islands (S3 Fig). Tide height ranged from 0.43 m to 1.63 m, as per Fiji Meteorological Service data.

### Characterization of levels of acoustic pressure

**Fig 3:**
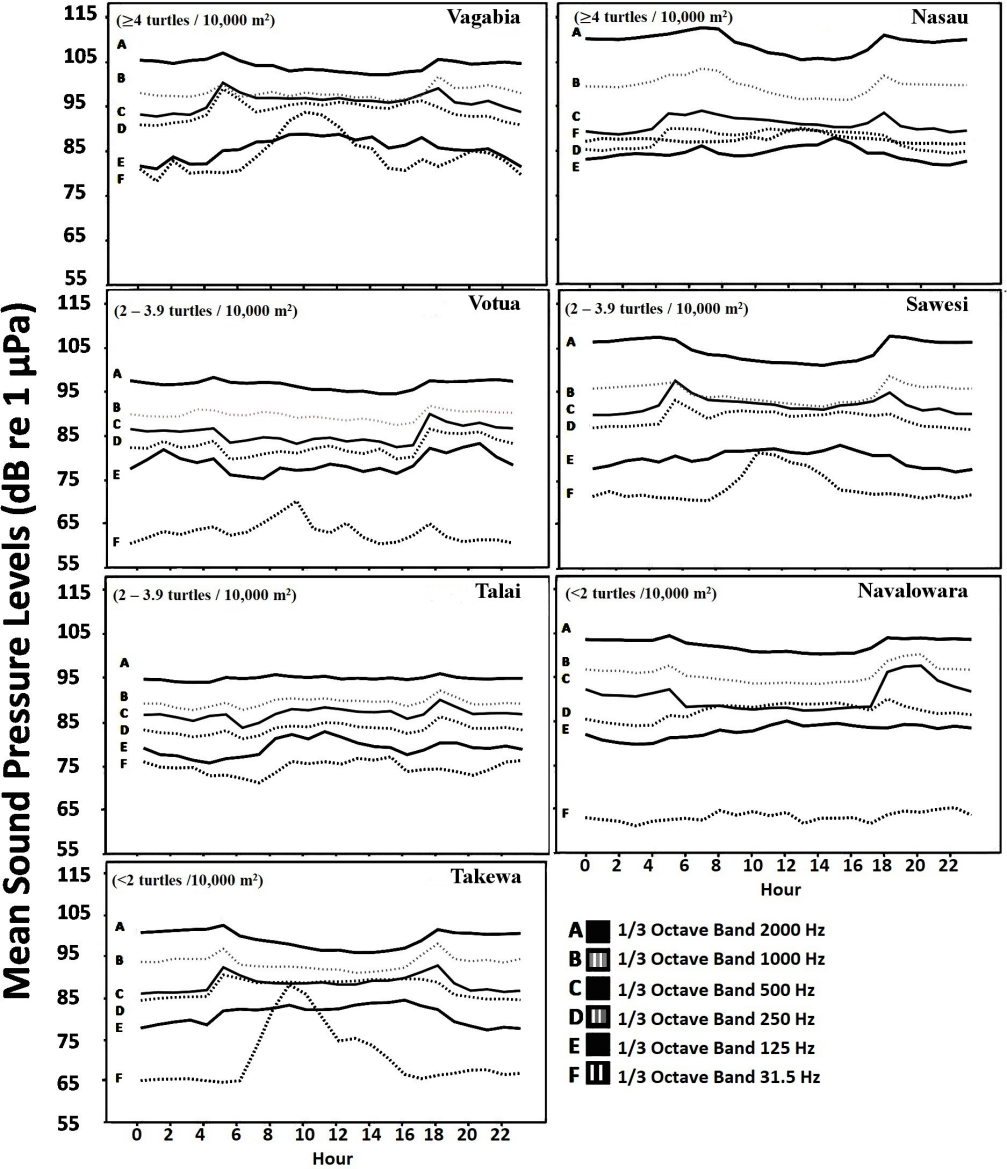
Circadian rhythm of the sound pressure levels at 31.5 Hz, 125 Hz, 250 Hz, 500 Hz, 1000 Hz, and 2000 Hz at the seven sites at Makogai Island and Yadua Island, Fiji, South Pacific. Sites are in descending order of turtle abundance, arranged horizontally from left to right. One site, Matauvia, is not presented, as the data collected were insufficient to evaluate the circadian rhythm.

A total of 31,118 min of recordings were collected. The mean and standard error of SPLs values measured at each site for the frequency bands centered from 16 Hz to 16 kHz are reported in S1 Table. The circadian rhythm for the frequency bands 31.5 Hz, 125 Hz, 250 Hz, 500 Hz, 1000 Hz, and 2000 Hz is reported for seven sites (Fig 3).

### Contribution of the acoustic components to the soundscape

Anthrophonies were identified in boat passages and detected in 0.25% of the 15,535 2-min files, which makes their contribution negligible. Therefore, no further analysis was conducted on this sound component. The SPLs centered at 31.5 Hz showed a cycle matching the daily tide increase for 86% of the sites, however, this was not significantly correlated at Talai (r_s_ = 0.01, p = 0.48). The SPLs centered at 125 Hz and 250 Hz were positively and significantly correlated with wind intensity (r_s_ = 0.37 to 0.65, p <0.001), except for Votua, for which very weak negative correlations were found (125 Hz: rs = –0.05, p <0.04; 250 Hz: rs = –0.06, p = 0.02). These SPLs presented a daily increase coincident with increasing wind intensity at both islands (S2 and S3 Figs). At the three subsequent band levels, i.e., 500 Hz, 1000 Hz, and 2000 Hz, a clear diel cycle emerged, with SPLs peaking at dusk and dawn (S3 Fig). These last three bands were especially influenced by the biological component, with fish impulsive signals at lower frequencies (band 500 Hz, positive Spearman correlation for all the sites: p <0.001), and crustacean snaps sounds at higher frequencies (1 kHz and 2 kHz, positive Spearman correlation for all the sites: p <0.001). Predictor variables *hours*, *fish* and *crustacean* explained from 23% to 92% of the GAM model deviance. In the best fitting GAM, the majority of the deviance was explained by fish sounds at 250 Hz and 500 Hz bands and crustacean sounds at 1 kHz and 2 kHz (S2 Table).

### Comparison among the soundscapes at the different sites

Average SPLs recorded at the eight sites along all the days (S1 Table) differed significantly at all the bands (Kruskal-Wallis test: 4,418 < χ^2^ < 12,723, p <0.001) (S3 Table). On average, SPL values were higher at two sites (Nasau and Vagabia) and lower at three sites (Matauvia, Takewa, and Votua), while moderate SPL values were measured at the remaining sites.

**Fig 4:**
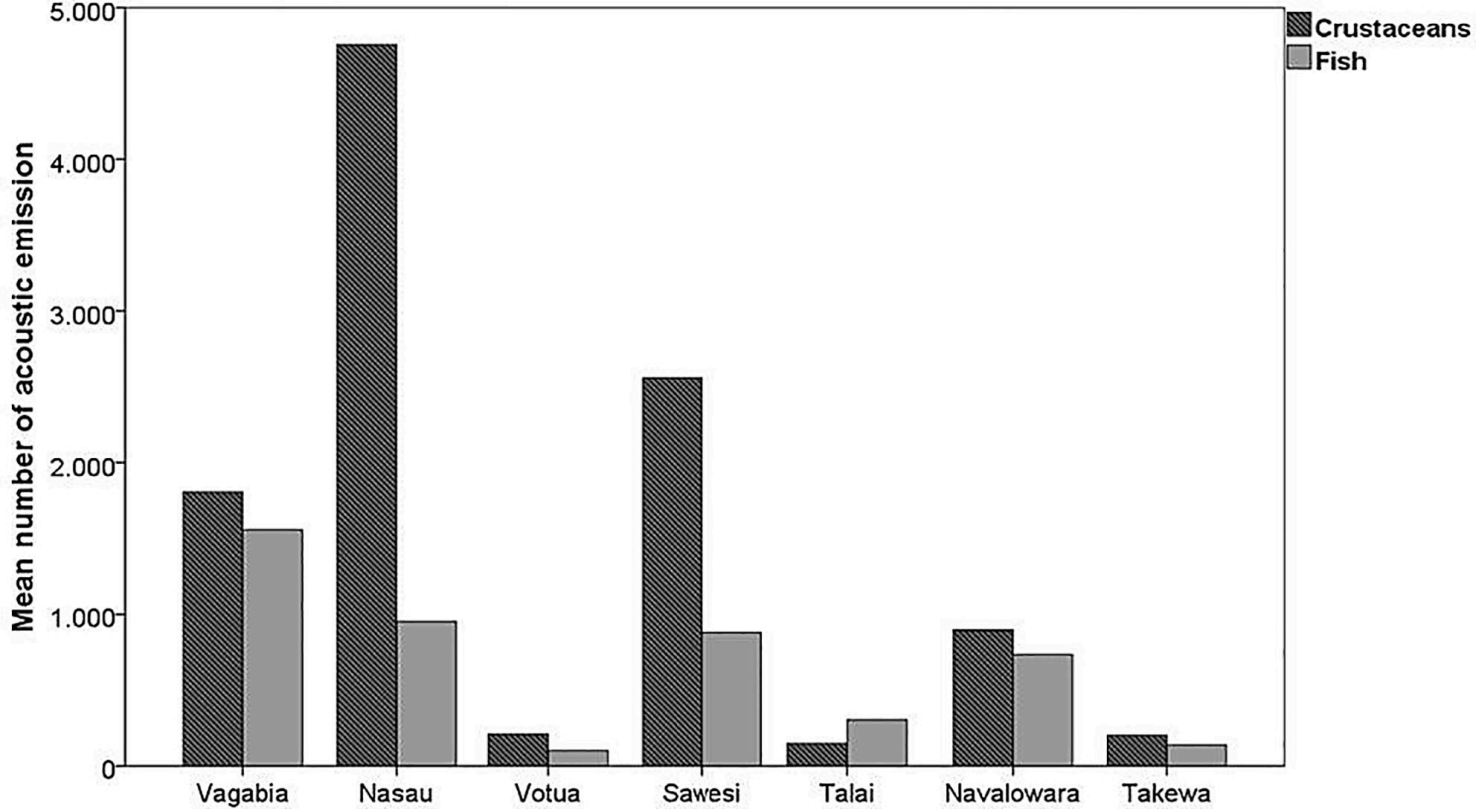
Plot of the mean number of acoustic emissions of fish and crustaceans at the seven green turtle foraging sites at Makogai Island and Yadua Island, Fiji, South Pacific, presented in order of turtle abundance.

At the frequency bands of 125–250 Hz, a difference of about 8–16 dB was recorded between the sites with the most and least exposure to wind stress. Regarding biophonies, acoustic emissions from fishes and crustaceans are graphically summarized in Fig 4. Overall, higher numbers of biological signals were recorded at three sites, two of which (Nasau and Vagabia) also showed higher SPL values and geophonies. Matauvia was excluded from the analysis, as the amount of data collected was insufficient to evaluate biological emissions.

## Discussion

This study documents the levels of acoustic pressure and the contribution of local biophonic and geophonic components to the overall soundscape of tropical neritic foraging habitats used by juvenile green turtles in a South Pacific island country. The soundscape can provide information about water turbulence under wind stress, which could inform sea turtles in their movements, and can provide information about habitat health through the intensity of local biological activity, a parameter that opportunistic green turtles might use to choose a feeding site.

Frequency bands observed in this study (16 Hz to 16 kHz) include the frequencies of green turtle electrophysiological response to underwater stimuli (50 Hz at 95 dB re: 1 μPa-rms to 1.6 kHz at 157 dB re: 1 μPa-rms, in [54]). The lower frequencies (<50 Hz) of the local soundscape were probably not perceived by the resident turtles; however, the effective hearing threshold for the juveniles of this species might be lower than what is known from auditory evoked potential measurements. Behavioral assessments, which are more sensitive than the auditory evoked potential for auditory threshold determination [60], could help to clarify this lower limit. The average level of energy of the local soundscape (86–102 dB re: 1 μPa-rms) observed at frequencies from 200 Hz to 700 Hz matched the hearing sensitivity of juvenile green turtles. Consequently, it is likely that the juvenile green turtles from different rookeries of the South Pacific and aggregating at the two Fijian islands [71] are capable of perceiving both the geophonic and biophonic components locally present.

The local soundscape was dominated by the sound generated by water turbulence such as due to breaking surf, tide, and wind-induced waves, that can be high energy and pervade low-frequency bands (31.5 to 125–250 Hz), especially during daylight hours. Higher frequency bands from 500 Hz can be associated with sonic fish choruses, emitted while in spawning aggregations or eventually defending territory and feeding, and by snapping shrimp clicks produced during aggressive behavior [88–90]. Their emissions show a clear diel pattern, as recorded elsewhere [12,44,43], with the highest peaks at dusk and dawn at all the seven sites.

Soundscape components are not static but change daily and seasonally, reflecting the temporal scale of the contribution from geophonies, biophonies, and anthrophonies. As these components ultimately depend on the condition of a particular habitat, they might be used by neritic-stage sea turtles for their daily navigation and orientation within coastal habitats that are relatively close. In particular, since physical forcings are among the main drivers of zooplankton dispersal [91], geophonic components like wind intensity might indirectly inform sea turtles about the presence of macro- and mega- zooplankton brought to the coast by higher wind intensity. The use of sensory stimuli by hard-shell sea turtles to orient towards their food choice has been proven chemically [92–94] and visually [95–97], and might extend to the use of acoustic stimuli. Comparison between geophonies, the abundance of planktonic food, and sea turtle spatial behavior recorded via satellite tracking would help clarify the effect of wind intensity on sea turtle behavior. Other geophonic components, such as those produced by the tides, might provide information towards site accessibility. In particular, they might inform about the best time to forage in coastal waters where the low tide would restrict turtle access to shallow subtidal areas, as well as guide turtle movements in the intertidal zone. Such tidal-dependent behavior, for example, was observed in green turtles in Australia and at the Comoro Islands, where the presence of turtles in seagrass beds and mangrove habitats was very low or null at low tides [98,99]. Similar behavior has been described for bottlenose dolphins (*Tursiops truncatus*), whose higher presence in shallow estuarine waters in Australia was recorded at high tide and possibly resulted from increased accessibility to prey items [100]. However, tidal currents can also be perceived mechanically, thus separating the influence of the two stimuli produced by the same event (tide) might be difficult. Playback experiments could help clarify the acoustic influence in absence of the mechanical stimulus.

The intensity of biophonic components like sounds produced by fishes and invertebrates, together with their diel cycle, might inform sea turtles about the overall quality of habitat, as well as likely abundance of possible benthic prey. The high number of fish and invertebrate signals recorded at some sites in this study provides a regular contribution to the local soundscape generating dusk and dawn choruses. This high-energy activity produces a marked increase in sound levels, especially at Vagabia and Nasau, the two sites of high turtle abundance. Accordingly, a lower number of biological emissions, such as recorded at Votua, Talai, and Takewa, contributed less to energy levels and therefore to lower SPL measures.

In this study, significant variation in the soundscape has been identified among the sites at the fine-scale level. Similar among-site variability was recorded on close coral reefs on the Great Barrier Reef, in Australia [34]. Our analyses revealed differences in temporal patterns and sound levels among bays, especially below 800 Hz. These differences resulted from the combination of biological signals and sounds generated by water turbulence. Green turtles occurred most frequently in the two sites with the highest SPLs, at all frequencies and throughout the day. The differences recorded among the average SPLs at the sites, and caused by biological and geophysics signals reached 18 dB re: 1 μPa. The two sites of high turtle abundance were characterized by higher acoustic energy.

Although variation was identified in the biotic and abiotic components observed at the different sites, no clear pattern explaining the different sea turtle abundances emerged, suggesting that several factors might have driven the turtle presence at a site. For example, one site of high turtle abundance (Vagabia) had relatively low marine plant coverage, while another site with relatively high seagrass coverage (Matauvia) had low turtle abundance. Seagrass meadows and algal beds have a patchy distribution in the two foraging islands, and given that green turtle diet in this area is largely made up of invertebrates [59], it is possible that green turtles were using acoustic cues and other stimuli to choose a specific feeding site within the broad foraging area. Consistent with this, very few turtles occurred where the soundscape indicated low water turbulence and low biological activity. Therefore, our results suggest that soundscape can contribute to informing sea turtles with critical details regarding each site, but whether the acoustic data are effectively used to actively select a site, or if they are used as proximate acoustic cues, lies outside the scope of this work. In addition, green turtles might use information acquired from their past experiences, such as the use of memory about long-term average conditions, as recently described for blue whales (*Balaenoptera musculus*) [101]. Playback experiments with the bands identified in this study should be used to investigate whether green turtle behavior is influenced by habitat soundscape while combining data recording of habitat soundscape with satellite tracking of sea turtles could provide evidence on the contribution of the habitat soundscape towards green turtle fine-scale movements among neritic habitats.

This work provides useful baseline soundscape information for habitats with relatively low anthropogenic acoustic impacts. An increase in ocean noise levels, especially along coastal areas, is a critical issue around the world [67]. High levels of noise spreading from anthropogenic activities have been recorded in a major sea turtle foraging area on the eastern coast of the USA [102]. The noise occupied the frequencies below 1 kHz, thus largely overlapping with the range of sea turtle hearing and, in particular, with the range of greater hearing sensitivity. Furthermore, green turtles displayed increased swimming speed and erratic behavior [103] when exposed to noise generated by pile driving, airguns, and sonar. Therefore, this study provides the first step in understanding the relevance of underwater natural soundscape on turtle abundance in a green turtle tropical neritic area and, as such, forms the basis for future conservation efforts in an age of increasing acoustic pollution in coastal waters.

## Supporting information

S1 Fig Underwater autonomous acoustic recorder. UAAR at Vagabia, in Makogai Island, Fiji. An iron 30 kg weight was used to anchor the UAAR, while a submerged buoy kept the mooring vertical.
(TIF)

S2 Fig Wind rose representing the distribution of wind directions and Beaufort wind intensity. (A) Makogai Island and (B) Yadua Island green turtle neritic foraging sites. Note: graphs have different scales.
(TIF)

S3 Fig Mean Beaufort wind intensity at Makogai Island and Yadua Island (Fiji, South Pacific) green turtle neritic foraging sites throughout the day. (TIF)

S1 Table Mean and standard error of the sound pressure levels at 1/3 octave bands centered from 16 Hz to 16 kHz dB re: 1 μPa-rms for each of the eight green turtle neritic foraging sites at Yadua and Makogai Islands, Fiji, South Pacific. (DOCX)

S2 Table Generalized additive model deviance. (DOCX)

S3 Table Results of post-hoc Tuckey test for average SPL analysis. (DOCX)
