# Supplementary figures and images for: Soundscape of green turtle foraging habitats in Fiji, South Pacific
Source: PLoS One. 2020 Aug 5;15(8):e0236628. doi: 10.1371/journal.pone.0236628 (PMC7406084; doi:10.1371/journal.pone.0236628)

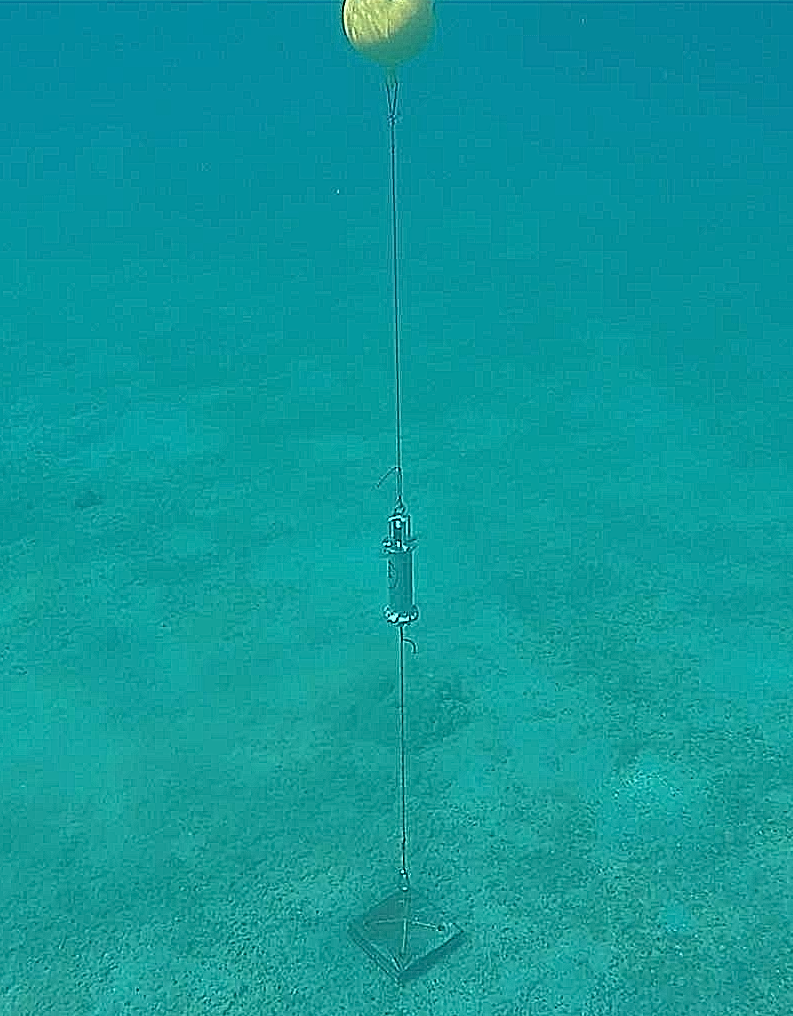

Supplement: S1 Fig — UAAR at Vagabia, in Makogai Island, Fiji. An iron 30 kg weight was used to anchor the UAAR, while a submerged buoy kept the mooring vertical. (TIF) [file pone.0236628.s001.TIF]

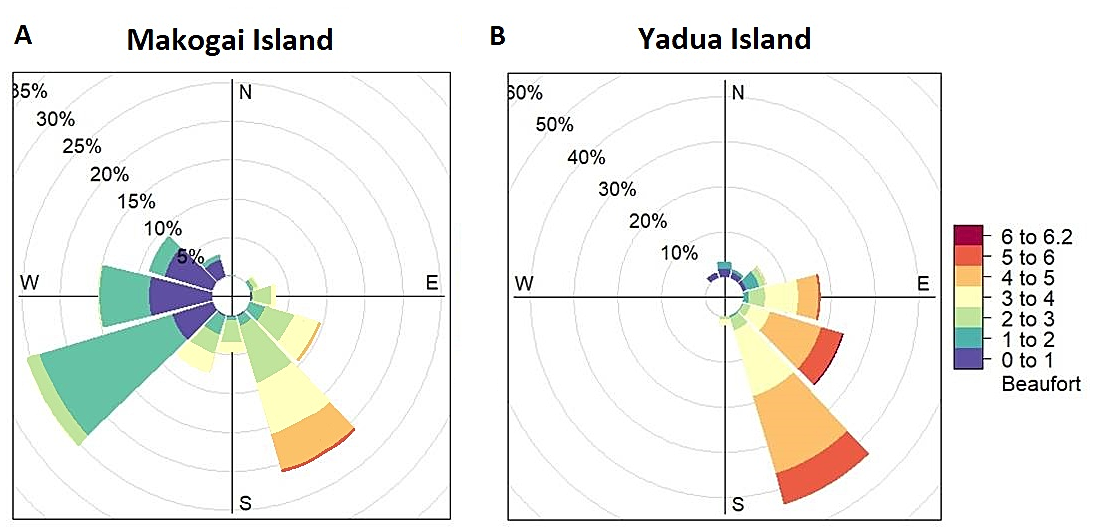

Supplement: S2 Fig — (A) Makogai Island and (B) Yadua Island green turtle neritic foraging sites. Note: graphs have different scales. (TIF) [file pone.0236628.s002.TIF]

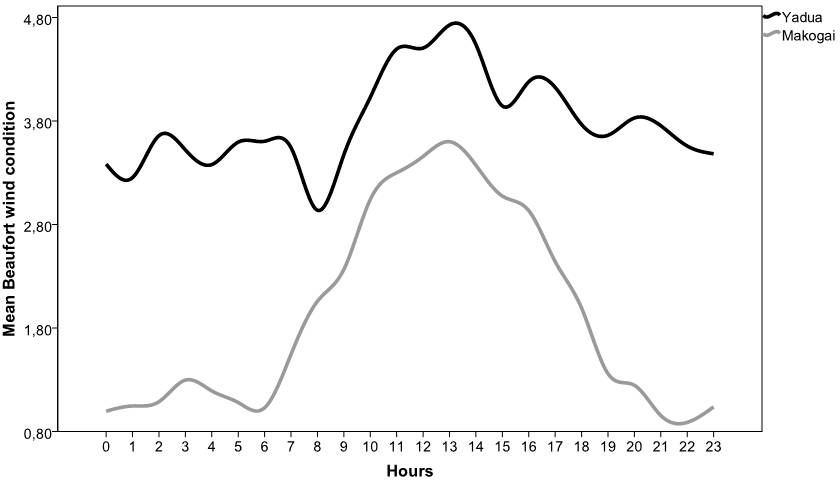

Supplement: S3 Fig — (TIF) [file pone.0236628.s003.TIF]
