# Supplementary material for: Soundscape of green turtle foraging habitats in Fiji, South Pacific
Source: PLoS One. 2020 Aug 5;15(8):e0236628. doi: 10.1371/journal.pone.0236628 (PMC7406084; doi:10.1371/journal.pone.0236628)
Supplement: S1 Table — (DOCX) [file pone.0236628.s004.docx]

**S1 Table. Mean and standard error of the sound pressure levels at 1/3 octave bands centered from 16 Hz to 16 kHz dB re: 1 µPa-rms for each of the eight green turtle neritic sites in Fiji, South Pacific.**

| *Central Frequency 1/3 Octave band (Hz)* | Makogai | | | | | | | | Yadua | | | | | | | |
| --- | --- | --- | --- | --- | --- | --- | --- | --- | --- | --- | --- | --- | --- | --- | --- | --- |
|  | Nasau | | Sawesi | | Takewa | | Vagabia | | Matauvia | | Navalowara | | Talai | | Votua | |
|  | Mean | SE | Mean | SE | Mean | SE | Mean | SE | Mean | SE | Mean | SE | Mean | SE | Mean | SE |
| **16** | 96.33 | 0.27 | 89.88 | 0.59 | 86.29 | 0.22 | 98.61 | 0.23 | 73.10 | 0.42 | 84.33 | 1.84 | 92.87 | 0.59 | 89.77 | 2.66 |
| **20** | 95.89 | 0.28 | 89.11 | 0.63 | 84.47 | 0.22 | 98.22 | 0.23 | 71.10 | 0.43 | 82.46 | 2.04 | 92.20 | 0.50 | 88.71 | 2.89 |
| **25** | 92.99 | 0.22 | 85.71 | 0.62 | 81.45 | 0.23 | 96.75 | 0.23 | 66.15 | 0.42 | 80.97 | 2.19 | 89.85 | 0.84 | 88.55 | 2.91 |
| **31.5** | 89.66 | 0.19 | 82.44 | 0.89 | 78.57 | 0.40 | 96.53 | 0.27 | 60.89 | 1.14 | 80.80 | 1.85 | 87.37 | 1.60 | 89.30 | 2.94 |
| **40** | 86.97 | 0.34 | 78.56 | 1.24 | 77.28 | 1.65 | 96.94 | 0.33 | 63.01 | 1.77 | 81.07 | 1.91 | 85.21 | 1.79 | 88.30 | 2.93 |
| **50** | 86.02 | 0.35 | 77.88 | 1.27 | 77.51 | 2.29 | 98.33 | 0.40 | 65.14 | 0.78 | 85.02 | 2.20 | 88.60 | 2.28 | 86.99 | 2.80 |
| **63** | 86.46 | 0.41 | 78.73 | 0.96 | 86.14 | 2.90 | 99.33 | 0.43 | 70.93 | 0.72 | 92.85 | 2.47 | 97.07 | 2.86 | 86.31 | 2.08 |
| **80** | 88.27 | 0.49 | 80.68 | 0.97 | 93.13 | 2.91 | 99.34 | 0.44 | 78.93 | 0.24 | 94.84 | 2.15 | 95.70 | 2.59 | 85.32 | 1.42 |
| **100** | 88.58 | 0.47 | 82.57 | 0.89 | 91.46 | 2.75 | 99.76 | 0.45 | 80.92 | 0.60 | 94.64 | 2.31 | 94.27 | 2.10 | 83.79 | 0.73 |
| **125** | 88.16 | 0.56 | 84.49 | 0.91 | 86.84 | 1.80 | 98.23 | 0.48 | 81.71 | 0.39 | 95.74 | 2.25 | 97.59 | 2.28 | 84.91 | 0.52 |
| **160** | 86.92 | 0.34 | 85.56 | 0.58 | 86.20 | 0.69 | 95.98 | 0.55 | 84.22 | 0.26 | 91.81 | 1.93 | 94.70 | 2.23 | 85.80 | 0.44 |
| **200** | 87.73 | 0.26 | 87.70 | 0.30 | 86.62 | 0.31 | 94.84 | 0.38 | 86.15 | 0.21 | 89.42 | 1.09 | 91.55 | 1.96 | 86.16 | 0.40 |
| **250** | 88.82 | 0.20 | 90.72 | 0.18 | 87.48 | 0.19 | 96.60 | 0.29 | 86.87 | 0.15 | 89.17 | 0.72 | 88.88 | 1.32 | 85.58 | 0.36 |
| **315** | 90.09 | 0.19 | 93.15 | 0.15 | 88.13 | 0.13 | 98.44 | 0.15 | 86.88 | 0.11 | 89.61 | 0.68 | 88.49 | 1.01 | 85.58 | 0.21 |
| **400** | 90.92 | 0.16 | 93.75 | 0.13 | 88.28 | 0.19 | 99.68 | 0.41 | 86.85 | 0.13 | 91.26 | 0.73 | 90.12 | 1.07 | 86.05 | 0.25 |
| **500** | 91.83 | 0.22 | 93.45 | 0.16 | 88.41 | 0.27 | 101.16 | 0.51 | 86.82 | 0.15 | 95.35 | 0.32 | 92.13 | 1.18 | 87.08 | 0.46 |
| **630** | 94.05 | 0.17 | 93.58 | 0.15 | 90.04 | 0.41 | 100.73 | 0.35 | 87.05 | 0.16 | 98.87 | 0.34 | 91.61 | 0.84 | 88.43 | 0.15 |
| **800** | 97.17 | 0.14 | 94.44 | 0.20 | 92.59 | 0.45 | 102.05 | 0.48 | 88.25 | 0.14 | 97.83 | 0.27 | 91.95 | 0.89 | 88.77 | 0.13 |
| **1000** | 100.21 | 0.11 | 96.17 | 0.21 | 93.58 | 0.29 | 105.88 | 0.73 | 89.30 | 0.19 | 97.99 | 0.17 | 96.37 | 1.74 | 90.56 | 0.41 |
| **1250** | 103.27 | 0.13 | 99.14 | 0.14 | 93.92 | 0.15 | 102.86 | 0.39 | 91.40 | 0.18 | 99.56 | 0.11 | 100.61 | 2.61 | 92.14 | 0.13 |
| **1600** | 106.42 | 0.11 | 102.71 | 0.07 | 96.07 | 0.08 | 103.45 | 0.08 | 94.43 | 0.18 | 101.36 | 0.05 | 96.68 | 1.80 | 94.52 | 0.04 |
| **2000** | 109.34 | 0.09 | 105.60 | 0.07 | 98.93 | 0.05 | 105.79 | 0.08 | 96.28 | 0.18 | 103.46 | 0.04 | 95.48 | 0.22 | 97.23 | 0.05 |
| **2500** | 111.71 | 0.05 | 108.33 | 0.05 | 102.43 | 0.06 | 107.47 | 0.07 | 98.38 | 0.17 | 105.63 | 0.04 | 97.36 | 0.12 | 99.62 | 0.04 |
| **3150** | 115.11 | 0.04 | 113.22 | 0.05 | 106.75 | 0.06 | 110.42 | 0.08 | 101.31 | 0.18 | 109.38 | 0.04 | 101.21 | 0.09 | 102.78 | 0.04 |
| **4000** | 113.30 | 0.04 | 112.29 | 0.05 | 103.20 | 0.05 | 109.53 | 0.11 | 99.19 | 0.15 | 108.19 | 0.04 | 99.77 | 0.08 | 101.00 | 0.04 |
| **5000** | 110.68 | 0.05 | 109.94 | 0.06 | 102.92 | 0.05 | 108.20 | 0.06 | 99.95 | 0.10 | 105.25 | 0.03 | 97.26 | 0.09 | 100.21 | 0.04 |
| **6300** | 113.03 | 0.05 | 114.49 | 0.06 | 105.84 | 0.04 | 110.91 | 0.06 | 102.97 | 0.09 | 109.27 | 0.03 | 99.56 | 0.06 | 103.14 | 0.03 |
| **8000** | 113.00 | 0.05 | 111.85 | 0.06 | 106.45 | 0.04 | 110.56 | 0.06 | 102.75 | 0.08 | 109.31 | 0.03 | 99.87 | 0.04 | 103.29 | 0.03 |
| **10000** | 112.21 | 0.05 | 110.49 | 0.08 | 106.04 | 0.04 | 109.93 | 0.06 | 101.89 | 0.08 | 108.98 | 0.03 | 98.99 | 0.03 | 102.65 | 0.03 |
| **12500** | 110.49 | 0.09 | 107.12 | 0.15 | 104.94 | 0.04 | 108.22 | 0.06 | 100.14 | 0.07 | 107.23 | 0.03 | 97.19 | 0.02 | 101.00 | 0.03 |
| **16000** | 109.58 | 0.11 | 106.64 | 0.18 | 102.89 | 0.04 | 107.77 | 0.05 | 98.65 | 0.10 | 106.07 | 0.03 | 95.18 | 0.03 | 100.07 | 0.03 |
