# Supplementary material for: Soundscape of green turtle foraging habitats in Fiji, South Pacific
Source: PLoS One. 2020 Aug 5;15(8):e0236628. doi: 10.1371/journal.pone.0236628 (PMC7406084; doi:10.1371/journal.pone.0236628)
Supplement: S2 Table — (DOCX) [file pone.0236628.s005.docx]

**S2 Table. Generalized additive model deviance.**

| **Site** | **Band** | **Variable** | **F value** | **P value** | **Deviance explained** |
| --- | --- | --- | --- | --- | --- |
| Vagabia | 125 | Hour | 57.895 | <0.001 | 48.0 % |
|  |  | Fish emissions | 3.922 | <0.001 |  |
|  |  | Crustacean emissions | 34.725 | <0.001 |  |
|  | 250 | Hour | 10.279 | <0.001 | 58.2 % |
|  |  | Fish emissions | 22.511 | <0.001 |  |
|  |  | Crustacean emissions | 8.331 | <0.001 |  |
|  | 500 | Hour | 21.59 | <0.001 | 55.7 % |
|  |  | Fish emissions | 14.08 | <0.001 |  |
|  |  | Crustacean emissions | 27.49 | <0.001 |  |
|  | 1000 | Hour | 23.084 | <0.001 | 39.8 % |
|  |  | Fish emissions | 2.988 | <0.001 |  |
|  |  | Crustaceans emissions | 35.217 | <0.001 |  |
|  | 2000 | Hour | 6.382 | <0.001 | 23.1 % |
|  |  | Fish emissions | 2.593 | <0.001 |  |
|  |  | Crustacean emissions | 6.575 | <0.001 |  |
| Nasau | 125 | Hour | 34.400 | <0.001 | 60.8 % |
|  |  | Fish emissions | 83.012 | <0.001 |  |
|  |  | Crustacean emissions | 7.157 | <0.001 |  |
|  | 250 | Hour | 56.773 | <0.001 | 72.1 % |
|  |  | Fish emissions | 41.075 | <0.001 |  |
|  |  | Crustacean emissions | 1.766 | <0.001 |  |
|  | 500 | Hour | 72.825 | <0.001 | 58.0 % |
|  |  | Fish emissions | 14.721 | <0.001 |  |
|  |  | Crustacean emissions | 2.598 | <0.001 |  |
|  | 1000 | Hour | 65.749 | <0.001 | 47.4 % |
|  |  | Fish emissions | 2.765 | <0.001 |  |
|  |  | Crustacean emissions | 4.529 | <0.001 |  |
|  | 2000 | Hour | 63.375 | <0.001 | 56.0 % |
|  |  | Fish emissions | 3.171 | <0.001 |  |
|  |  | Crustacean emissions | 7.660 | <0.001 |  |
| Talei | 125 | Hour | 26.215 | <0.001 | 62.3 % |
|  |  | Fish emissions | 82.064 | <0.001 |  |
|  |  | Crustacean emissions | 0.679 | <0.001 |  |
|  | 250 | Hour | 30.773 | <0.001 | 81.4 % |
|  |  | Fish emissions | 276.861 | <0.001 |  |
|  |  | Crustacean emissions | 2.159 | <0.001 |  |
|  | 500 | Hour | 47.00 | <0.001 | 84.2 % |
|  |  | Fish emissions | 318.59 | <0.001 |  |
|  |  | Crustacean emissions | 2.16 | <0.001 |  |
|  | 1000 | Hour | 31.82 | <0.001 | 74.5% |
|  |  | Fish emissions | 111.17 | <0.001 |  |
|  |  | Crustacean emissions | 12.80 | <0.001 |  |
|  | 2000 | Hour | 9.884 | <0.001 | 56.2% |
|  |  | Fish emissions | 8.587 | <0.001 |  |
|  |  | Crustacean emissions | 39.168 | <0.001 |  |
| Votua | 125 | Hour | 16.684 | <0.001 | 52.5% |
|  |  | Fish emissions | 47.366 | <0.001 |  |
|  |  | Crustacean emissions | 4.201 | <0.001 |  |
|  | 250 | Hour | 26.811 | <0.001 | 69.1% |
|  |  | Fish emissions | 100.859 | <0.001 |  |
|  |  | Crustacean emissions | 3.518 | <0.001 |  |
|  | 500 | Hour | 10.096 | <0.001 | 69.5% |
|  |  | Fish emissions | 67.173 | <0.001 |  |
|  |  | Crustacean emissions | 4.024 | <0.001 |  |
|  | 1000 | Hour | 17.356 | <0.001 | 43.0% |
|  |  | Fish emissions | 6.705 | <0.001 |  |
|  |  | Crustacean emissions | 9.390 | <0.001 |  |
|  | 2000 | Hour | 30.98 | <0.001 | 62.9% |
|  |  | Fish emissions | 1.23 | <0.001 |  |
|  |  | Crustacean emissions | 42.25 | <0.001 |  |
| Savesi | 125 | Hour | 22.716 | <0.001 | 38.5% |
|  |  | Fish emissions | 7.095 | <0.001 |  |
|  |  | Crustacean emissions | 2.094 | <0.001 |  |
|  | 250 | Hour | 16.71 | <0.001 | 63.9% |
|  |  | Fish emissions | 67.36 | <0.001 |  |
|  |  | Crustacean emissions | 14.31 | <0.001 |  |
|  | 500 | Hour | 92.254 | <0.001 | 77.1% |
|  |  | Fish emissions | 75.699 | <0.001 |  |
|  |  | Crustacean emissions | 6.288 | <0.001 |  |
|  | 1000 | Hour | 31.767 | <0.001 | 76.5% |
|  |  | Fish emissions | 1.439 | <0.001 |  |
|  |  | Crustacean emissions | 33.484 | <0.001 |  |
|  | 2000 | Hour | 81.818 | <0.001 | 92.2% |
|  |  | Fish emissions | 9.885 | <0.001 |  |
|  |  | Crustacean emissions | 59.522 | <0.001 |  |
| Navalowara | 125 | Hour | 94.681 | <0.001 | 45.7% |
|  |  | Fish emissions | 13.391 | <0.001 |  |
|  |  | Crustacean emissions | 0.972 | <0.001 |  |
|  | 250 | Hour | 246.433 | <0.001 | 73.4% |
|  |  | Fish emissions | 22.443 | <0.001 |  |
|  |  | Crustacean emissions | 4.371 | <0.001 |  |
|  | 500 | Hour | 80.72 | <0.001 | 88.6% |
|  |  | Fish emissions | 21.07 | <0.001 |  |
|  |  | Crustacean emissions | 55.48 | <0.001 |  |
|  | 1000 | Hour | 14.784 | <0.001 | 84.6% |
|  |  | Fish emissions | 6.231 | <0.001 |  |
|  |  | Crustacean emissions | 65.952 | <0.001 |  |
|  | 2000 | Hour | 3.378 | <0.001 | 78.2% |
|  |  | Fish emissions | 3.027 | <0.001 |  |
|  |  | Crustacean emissions | 78.949 | <0.001 |  |
| Takewa | 125 | Hour | 15.651 | <0.001 | 43.6% |
|  |  | Fish emissions | 22.962 | <0.001 |  |
|  |  | Crustacean emissions | 0.641 | <0.001 |  |
|  | 250 | Hour | 28.105 | <0.001 | 75.3% |
|  |  | Fish emissions | 90.758 | <0.001 |  |
|  |  | Crustacean emissions | 0.774 | 0.01 |  |
|  | 500 | Hour | 117.597 | <0.001 | 77.5% |
|  |  | Fish emissions | 74.925 | <0.001 |  |
|  |  | Crustacean emissions | 9.338 | <0.001 |  |
|  | 1000 | Hour | 53.854 | <0.001 | 58.7% |
|  |  | Fish emissions | 3.499 | <0.001 |  |
|  |  | Crustacean emissions | 31.953 | <0.001 |  |
|  | 2000 | Hour | 111.166 | <0.001 | 89.4% |
|  |  | Fish emissions | 0.572 | <0.001 |  |
|  |  | Crustacean emissions | 86.104 | <0.001 |  |
