# Supplementary material for: Soundscape of green turtle foraging habitats in Fiji, South Pacific
Source: PLoS One. 2020 Aug 5;15(8):e0236628. doi: 10.1371/journal.pone.0236628 (PMC7406084; doi:10.1371/journal.pone.0236628)
Supplement: S3 Table — (DOCX) [file pone.0236628.s006.docx]

**S3 Table. Results of post-hoc Tuckey test for average SPL analysis.**

| Tuckey test | | | | | |
| --- | --- | --- | --- | --- | --- |
| Band | Embayment | | Difference among means | Standard Error | Sig. |
| 16 Hz | Nasau | Navalowara | 17,84 | 0,21 | **<0,001** |
|  |  | Savesi | 11,58 | 0,21 | **<0,001** |
|  |  | Takewa | 14,24 | 0,20 | **<0,001** |
|  |  | Vagabia | 4,32 | 0,20 | **<0,001** |
|  |  | Matauvia | 19,88 | 1,01 | **<0,001** |
|  |  | Talai | 9,02 | 0,20 | **<0,001** |
|  |  | Votua | 18,86 | 0,23 | **<0,001** |
|  | Navalowara | Savesi | -6,26 | 0,20 | **<0,001** |
|  |  | Takewa | -3,60 | 0,19 | **<0,001** |
|  |  | Vagabia | -13,52 | 0,19 | **<0,001** |
|  |  | Matauvia | 2,04 | 1,00 | 0,459 |
|  |  | Talai | -8,83 | 0,19 | **<0,001** |
|  |  | Votua | 1,02 | 0,21 | **<0,001** |
|  | Savesi | Takewa | 2,66 | 0,20 | **<0,001** |
|  |  | Vagabia | -7,26 | 0,20 | **<0,001** |
|  |  | Matauvia | 8,30 | 1,00 | **<0,001** |
|  |  | Talai | -2,57 | 0,19 | **<0,001** |
|  |  | Votua | 7,28 | 0,22 | **<0,001** |
|  | Takewa | Vagabia | -9,92 | 0,19 | **<0,001** |
|  |  | Matauvia | 5,64 | 1,00 | **<0,001** |
|  |  | Talai | -5,23 | 0,18 | **<0,001** |
|  |  | Votua | 4,62 | 0,21 | **<0,001** |
|  | Vagabia | Matauvia | 15,56 | 1,00 | **<0,001** |
|  |  | Talai | 4,69 | 0,18 | **<0,001** |
|  |  | Votua | 14,54 | 0,21 | **<0,001** |
|  | Matauvia | Talai | -10,87 | 1,00 | **<0,001** |
|  |  | Votua | -1,02 | 1,01 | 0,973 |
|  | Talai | Votua | 9,85 | 0,21 | **<0,001** |
| 20 Hz | Nasau | Navalowara | 18,93 | 0,21 | **<0,001** |
|  |  | Savesi | 12,26 | 0,22 | **<0,001** |
|  |  | Takewa | 15,19 | 0,21 | **<0,001** |
|  |  | Vagabia | 4,30 | 0,21 | **<0,001** |
|  |  | Matauvia | 21,31 | 1,02 | **<0,001** |
|  |  | Talai | 9,66 | 0,20 | **<0,001** |
|  |  | Votua | 20,23 | 0,23 | **<0,001** |
|  | Navalowara | Savesi | -6,67 | 0,20 | **<0,001** |
|  |  | Takewa | -3,73 | 0,19 | **<0,001** |
|  |  | Vagabia | -14,62 | 0,19 | **<0,001** |
|  |  | Matauvia | 2,38 | 1,02 | 0,271 |
|  |  | Talai | -9,27 | 0,19 | **<0,001** |
|  |  | Votua | 1,30 | 0,21 | **<0,001** |
|  | Savesi | Takewa | 2,93 | 0,20 | **<0,001** |
|  |  | Vagabia | -7,96 | 0,20 | **<0,001** |
|  |  | Matauvia | 9,05 | 1,02 | **<0,001** |
|  |  | Talai | -2,60 | 0,20 | **<0,001** |
|  |  | Votua | 7,97 | 0,22 | **<0,001** |
|  | Takewa | Vagabia | -10,89 | 0,19 | **<0,001** |
|  |  | Matauvia | 6,12 | 1,02 | **<0,001** |
|  |  | Talai | -5,53 | 0,19 | **<0,001** |
|  |  | Votua | 5,04 | 0,21 | **<0,001** |
|  | Vagabia | Matauvia | 17,01 | 1,02 | **<0,001** |
|  |  | Talai | 5,36 | 0,19 | **<0,001** |
|  |  | Votua | 15,92 | 0,21 | **<0,001** |
|  | Matauvia | Talai | -11,65 | 1,02 | **<0,001** |
|  |  | Votua | -1,08 | 1,02 | 0,965 |
|  | Talai | Votua | 10,57 | 0,21 | **<0,001** |
| 25 Hz | Nasau | Navalowara | 21,36 | 0,23 | **<0,001** |
|  |  | Savesi | 13,37 | 0,23 | **<0,001** |
|  |  | Takewa | 17,14 | 0,22 | **<0,001** |
|  |  | Vagabia | 4,41 | 0,22 | **<0,001** |
|  |  | Matauvia | 24,23 | 1,10 | **<0,001** |
|  |  | Talai | 11,08 | 0,22 | **<0,001** |
|  |  | Votua | 22,67 | 0,25 | **<0,001** |
|  | Navalowara | Savesi | -7,99 | 0,22 | **<0,001** |
|  |  | Takewa | -4,22 | 0,21 | **<0,001** |
|  |  | Vagabia | -16,95 | 0,21 | **<0,001** |
|  |  | Matauvia | 2,86 | 1,10 | 0,151 |
|  |  | Talai | -10,28 | 0,20 | **<0,001** |
|  |  | Votua | 1,30 | 0,23 | **<0,001** |
|  | Savesi | Takewa | 3,77 | 0,21 | **<0,001** |
|  |  | Vagabia | -8,96 | 0,22 | **<0,001** |
|  |  | Matauvia | 10,85 | 1,10 | **<0,001** |
|  |  | Talai | -2,29 | 0,21 | **<0,001** |
|  |  | Votua | 9,30 | 0,24 | **<0,001** |
|  | Takewa | Vagabia | -12,73 | 0,20 | **<0,001** |
|  |  | Matauvia | 7,08 | 1,09 | **<0,001** |
|  |  | Talai | -6,06 | 0,20 | **<0,001** |
|  |  | Votua | 5,53 | 0,23 | **<0,001** |
|  | Vagabia | Matauvia | 19,82 | 1,10 | **<0,001** |
|  |  | Talai | 6,67 | 0,20 | **<0,001** |
|  |  | Votua | 18,26 | 0,23 | **<0,001** |
|  | Matauvia | Talai | -13,14 | 1,09 | **<0,001** |
|  |  | Votua | -1,56 | 1,10 | 0,850 |
|  | Talai | Votua | 11,59 | 0,23 | **<0,001** |
| 31.5 Hz | Nasau | Navalowara | 25,30 | 0,24 | **<0,001** |
|  |  | Savesi | 13,50 | 0,25 | **<0,001** |
|  |  | Takewa | 18,98 | 0,24 | **<0,001** |
|  |  | Vagabia | 3,34 | 0,24 | **<0,001** |
|  |  | Matauvia | 29,81 | 1,19 | **<0,001** |
|  |  | Talai | 13,01 | 0,24 | **<0,001** |
|  |  | Votua | 26,28 | 0,27 | **<0,001** |
|  | Navalowara | Savesi | -11,80 | 0,24 | **<0,001** |
|  |  | Takewa | -6,31 | 0,22 | **<0,001** |
|  |  | Vagabia | -21,96 | 0,22 | **<0,001** |
|  |  | Matauvia | 4,51 | 1,18 | **<0,001** |
|  |  | Talai | -12,29 | 0,22 | **<0,001** |
|  |  | Votua | 0,98 | 0,25 | **<0,001** |
|  | Savesi | Takewa | 5,49 | 0,23 | **<0,001** |
|  |  | Vagabia | -10,16 | 0,23 | **<0,001** |
|  |  | Matauvia | 16,31 | 1,18 | **<0,001** |
|  |  | Talai | -0,49 | 0,23 | 0,391 |
|  |  | Votua | 12,79 | 0,26 | **<0,001** |
|  | Takewa | Vagabia | -15,64 | 0,22 | **<0,001** |
|  |  | Matauvia | 10,82 | 1,18 | **<0,001** |
|  |  | Talai | -5,98 | 0,22 | **<0,001** |
|  |  | Votua | 7,30 | 0,25 | **<0,001** |
|  | Vagabia | Matauvia | 26,47 | 1,18 | **<0,001** |
|  |  | Talai | 9,67 | 0,22 | **<0,001** |
|  |  | Votua | 22,94 | 0,25 | **<0,001** |
|  | Matauvia | Talai | -16,80 | 1,18 | **<0,001** |
|  |  | Votua | -3,52 | 1,19 | 0,060 |
|  | Talai | Votua | 13,28 | 0,24 | **<0,001** |
| 40 Hz | Nasau | Navalowara | 24,66 | 0,26 | **<0,001** |
|  |  | Savesi | 13,42 | 0,27 | **<0,001** |
|  |  | Takewa | 20,39 | 0,25 | **<0,001** |
|  |  | Vagabia | 2,86 | 0,26 | **<0,001** |
|  |  | Matauvia | 29,95 | 1,25 | **<0,001** |
|  |  | Talai | 14,45 | 0,25 | **<0,001** |
|  |  | Votua | 27,50 | 0,28 | **<0,001** |
|  | Navalowara | Savesi | -11,24 | 0,25 | **<0,001** |
|  |  | Takewa | -4,27 | 0,23 | **<0,001** |
|  |  | Vagabia | -21,79 | 0,24 | **<0,001** |
|  |  | Matauvia | 5,29 | 1,25 | **<0,001** |
|  |  | Talai | -10,21 | 0,23 | **<0,001** |
|  |  | Votua | 2,84 | 0,26 | **<0,001** |
|  | Savesi | Takewa | 6,97 | 0,24 | **<0,001** |
|  |  | Vagabia | -10,56 | 0,25 | **<0,001** |
|  |  | Matauvia | 16,53 | 1,25 | **<0,001** |
|  |  | Talai | 1,03 | 0,24 | **<0,001** |
|  |  | Votua | 14,08 | 0,27 | **<0,001** |
|  | Takewa | Vagabia | -17,53 | 0,23 | **<0,001** |
|  |  | Matauvia | 9,56 | 1,25 | **<0,001** |
|  |  | Talai | -5,94 | 0,23 | **<0,001** |
|  |  | Votua | 7,11 | 0,26 | **<0,001** |
|  | Vagabia | Matauvia | 27,09 | 1,25 | **<0,001** |
|  |  | Talai | 11,59 | 0,23 | **<0,001** |
|  |  | Votua | 24,64 | 0,26 | **<0,001** |
|  | Matauvia | Talai | -15,50 | 1,25 | **<0,001** |
|  |  | Votua | -2,45 | 1,25 | 0,515 |
|  | Talai | Votua | 13,05 | 0,26 | **<0,001** |
| 50 Hz | Nasau | Navalowara | 17,86 | 0,24 | **<0,001** |
|  |  | Savesi | 12,17 | 0,25 | **<0,001** |
|  |  | Takewa | 19,81 | 0,24 | **<0,001** |
|  |  | Vagabia | 2,66 | 0,24 | **<0,001** |
|  |  | Matauvia | 22,64 | 1,17 | **<0,001** |
|  |  | Talai | 14,94 | 0,24 | **<0,001** |
|  |  | Votua | 23,53 | 0,26 | **<0,001** |
|  | Navalowara | Savesi | -5,69 | 0,23 | **<0,001** |
|  |  | Takewa | 1,95 | 0,22 | **<0,001** |
|  |  | Vagabia | -15,20 | 0,22 | **<0,001** |
|  |  | Matauvia | 4,78 | 1,17 | **<0,001** |
|  |  | Talai | -2,92 | 0,22 | **<0,001** |
|  |  | Votua | 5,67 | 0,25 | **<0,001** |
|  | Savesi | Takewa | 7,64 | 0,23 | **<0,001** |
|  |  | Vagabia | -9,51 | 0,23 | **<0,001** |
|  |  | Matauvia | 10,48 | 1,17 | **<0,001** |
|  |  | Talai | 2,77 | 0,23 | **<0,001** |
|  |  | Votua | 11,36 | 0,26 | **<0,001** |
|  | Takewa | Vagabia | -17,15 | 0,22 | **<0,001** |
|  |  | Matauvia | 2,84 | 1,17 | 0,229 |
|  |  | Talai | -4,87 | 0,21 | **<0,001** |
|  |  | Votua | 3,72 | 0,24 | **<0,001** |
|  | Vagabia | Matauvia | 19,98 | 1,17 | **<0,001** |
|  |  | Talai | 12,27 | 0,22 | **<0,001** |
|  |  | Votua | 20,87 | 0,25 | **<0,001** |
|  | Matauvia | Talai | -7,71 | 1,17 | **<0,001** |
|  |  | Votua | 0,88 | 1,18 | 0,995 |
|  | Talai | Votua | 8,59 | 0,24 | **<0,001** |
| 63 Hz | Nasau | Navalowara | 11,12 | 0,22 | **<0,001** |
|  |  | Savesi | 10,67 | 0,23 | **<0,001** |
|  |  | Takewa | 17,66 | 0,21 | **<0,001** |
|  |  | Vagabia | 2,20 | 0,22 | **<0,001** |
|  |  | Matauvia | 15,31 | 1,06 | **<0,001** |
|  |  | Talai | 14,24 | 0,21 | **<0,001** |
|  |  | Votua | 17,81 | 0,24 | **<0,001** |
|  | Navalowara | Savesi | -0,45 | 0,21 | 0,408 |
|  |  | Takewa | 6,54 | 0,20 | **<0,001** |
|  |  | Vagabia | -8,92 | 0,20 | **<0,001** |
|  |  | Matauvia | 4,19 | 1,06 | **<0,001** |
|  |  | Talai | 3,12 | 0,20 | **<0,001** |
|  |  | Votua | 6,69 | 0,22 | **<0,001** |
|  | Savesi | Takewa | 6,99 | 0,21 | **<0,001** |
|  |  | Vagabia | -8,48 | 0,21 | **<0,001** |
|  |  | Matauvia | 4,64 | 1,06 | **<0,001** |
|  |  | Talai | 3,56 | 0,21 | **<0,001** |
|  |  | Votua | 7,14 | 0,23 | **<0,001** |
|  | Takewa | Vagabia | -15,47 | 0,20 | **<0,001** |
|  |  | Matauvia | -2,35 | 1,06 | 0,341 |
|  |  | Talai | -3,42 | 0,19 | **<0,001** |
|  |  | Votua | 0,15 | 0,22 | 0,997 |
|  | Vagabia | Matauvia | 13,11 | 1,06 | **<0,001** |
|  |  | Talai | 12,04 | 0,20 | **<0,001** |
|  |  | Votua | 15,62 | 0,22 | **<0,001** |
|  | Matauvia | Talai | -1,07 | 1,06 | 0,973 |
|  |  | Votua | 2,50 | 1,07 | 0,267 |
|  | Talai | Votua | 3,58 | 0,22 | **<0,001** |
| 80 Hz | Nasau | Navalowara | 7,54 | 0,20 | **<0,001** |
|  |  | Savesi | 9,35 | 0,20 | **<0,001** |
|  |  | Takewa | 13,78 | 0,19 | **<0,001** |
|  |  | Vagabia | 1,93 | 0,19 | **<0,001** |
|  |  | Matauvia | 5,72 | 0,95 | **<0,001** |
|  |  | Talai | 12,14 | 0,19 | **<0,001** |
|  |  | Votua | 13,21 | 0,21 | **<0,001** |
|  | Navalowara | Savesi | 1,81 | 0,19 | **<0,001** |
|  |  | Takewa | 6,24 | 0,18 | **<0,001** |
|  |  | Vagabia | -5,62 | 0,18 | **<0,001** |
|  |  | Matauvia | -1,82 | 0,95 | 0,537 |
|  |  | Talai | 4,60 | 0,18 | **<0,001** |
|  |  | Votua | 5,67 | 0,20 | **<0,001** |
|  | Savesi | Takewa | 4,43 | 0,19 | **<0,001** |
|  |  | Vagabia | -7,43 | 0,19 | **<0,001** |
|  |  | Matauvia | -3,64 | 0,95 | **<0,001** |
|  |  | Talai | 2,79 | 0,18 | **<0,001** |
|  |  | Votua | 3,86 | 0,21 | **<0,001** |
|  | Takewa | Vagabia | -11,86 | 0,18 | **<0,001** |
|  |  | Matauvia | -8,06 | 0,95 | **<0,001** |
|  |  | Talai | -1,64 | 0,17 | **<0,001** |
|  |  | Votua | -0,57 | 0,20 | 0,073 |
|  | Vagabia | Matauvia | 3,79 | 0,95 | **<0,001** |
|  |  | Talai | 10,22 | 0,18 | **<0,001** |
|  |  | Votua | 11,28 | 0,20 | **<0,001** |
|  | Matauvia | Talai | 6,42 | 0,95 | **<0,001** |
|  |  | Votua | 7,49 | 0,95 | **<0,001** |
|  | Talai | Votua | 1,07 | 0,20 | **<0,001** |
| 100 Hz | Nasau | Navalowara | 4,72 | 0,18 | **<0,001** |
|  |  | Savesi | 6,61 | 0,18 | **<0,001** |
|  |  | Takewa | 8,65 | 0,17 | **<0,001** |
|  |  | Vagabia | 0,57 | 0,17 | **<0,001** |
|  |  | Matauvia | 4,56 | 0,85 | **<0,001** |
|  |  | Talai | 9,13 | 0,17 | **<0,001** |
|  |  | Votua | 9,53 | 0,19 | **<0,001** |
|  | Navalowara | Savesi | 1,89 | 0,17 | **<0,001** |
|  |  | Takewa | 3,93 | 0,16 | **<0,001** |
|  |  | Vagabia | -4,15 | 0,16 | **<0,001** |
|  |  | Matauvia | -0,16 | 0,85 | 1,000 |
|  |  | Talai | 4,41 | 0,16 | **<0,001** |
|  |  | Votua | 4,81 | 0,18 | **<0,001** |
|  | Savesi | Takewa | 2,04 | 0,17 | **<0,001** |
|  |  | Vagabia | -6,04 | 0,17 | **<0,001** |
|  |  | Matauvia | -2,05 | 0,85 | 0,237 |
|  |  | Talai | 2,52 | 0,16 | **<0,001** |
|  |  | Votua | 2,92 | 0,19 | **<0,001** |
|  | Takewa | Vagabia | -8,08 | 0,16 | **<0,001** |
|  |  | Matauvia | -4,09 | 0,85 | **<0,001** |
|  |  | Talai | 0,47 | 0,16 | **<0,001** |
|  |  | Votua | 0,88 | 0,18 | **<0,001** |
|  | Vagabia | Matauvia | 3,99 | 0,85 | **<0,001** |
|  |  | Talai | 8,56 | 0,16 | **<0,001** |
|  |  | Votua | 8,96 | 0,18 | **<0,001** |
|  | Matauvia | Talai | 4,57 | 0,85 | **<0,001** |
|  |  | Votua | 4,97 | 0,85 | **<0,001** |
|  | Talai | Votua | 0,41 | 0,18 | 0,287 |
| 125 Hz | Nasau | Navalowara | 1,83 | 0,15 | **<0,001** |
|  |  | Savesi | 3,71 | 0,16 | **<0,001** |
|  |  | Takewa | 4,84 | 0,15 | **<0,001** |
|  |  | Vagabia | -1,51 | 0,15 | **<0,001** |
|  |  | Matauvia | 3,58 | 0,73 | **<0,001** |
|  |  | Talai | 5,47 | 0,15 | **<0,001** |
|  |  | Votua | 6,24 | 0,16 | **<0,001** |
|  | Navalowara | Savesi | 1,88 | 0,15 | **<0,001** |
|  |  | Takewa | 3,01 | 0,14 | **<0,001** |
|  |  | Vagabia | -3,34 | 0,14 | **<0,001** |
|  |  | Matauvia | 1,75 | 0,73 | 0,239 |
|  |  | Talai | 3,65 | 0,14 | **<0,001** |
|  |  | Votua | 4,41 | 0,15 | **<0,001** |
|  | Savesi | Takewa | 1,13 | 0,14 | **<0,001** |
|  |  | Vagabia | -5,22 | 0,14 | **<0,001** |
|  |  | Matauvia | -0,13 | 0,73 | 1,000 |
|  |  | Talai | 1,76 | 0,14 | **<0,001** |
|  |  | Votua | 2,53 | 0,16 | **<0,001** |
|  | Takewa | Vagabia | -6,35 | 0,14 | **<0,001** |
|  |  | Matauvia | -1,26 | 0,73 | 0,674 |
|  |  | Talai | 0,64 | 0,13 | **<0,001** |
|  |  | Votua | 1,40 | 0,15 | **<0,001** |
|  | Vagabia | Matauvia | 5,09 | 0,73 | **<0,001** |
|  |  | Talai | 6,99 | 0,13 | **<0,001** |
|  |  | Votua | 7,75 | 0,15 | **<0,001** |
|  | Matauvia | Talai | 1,89 | 0,73 | 0,158 |
|  |  | Votua | 2,65 | 0,73 | **<0,001** |
|  | Talai | Votua | 0,76 | 0,15 | **<0,001** |
| 160 Hz | Nasau | Navalowara | -0,24 | 0,12 | 0,530 |
|  |  | Savesi | 1,30 | 0,13 | **<0,001** |
|  |  | Takewa | 2,32 | 0,12 | **<0,001** |
|  |  | Vagabia | -3,57 | 0,12 | **<0,001** |
|  |  | Matauvia | 0,99 | 0,60 | 0,727 |
|  |  | Talai | 3,64 | 0,12 | **<0,001** |
|  |  | Votua | 4,10 | 0,14 | **<0,001** |
|  | Navalowara | Savesi | 1,54 | 0,12 | **<0,001** |
|  |  | Takewa | 2,56 | 0,11 | **<0,001** |
|  |  | Vagabia | -3,33 | 0,11 | **<0,001** |
|  |  | Matauvia | 1,23 | 0,60 | 0,454 |
|  |  | Talai | 3,88 | 0,11 | **<0,001** |
|  |  | Votua | 4,34 | 0,13 | **<0,001** |
|  | Savesi | Takewa | 1,02 | 0,12 | **<0,001** |
|  |  | Vagabia | -4,87 | 0,12 | **<0,001** |
|  |  | Matauvia | -0,31 | 0,60 | 1,000 |
|  |  | Talai | 2,34 | 0,12 | **<0,001** |
|  |  | Votua | 2,80 | 0,13 | **<0,001** |
|  | Takewa | Vagabia | -5,89 | 0,11 | **<0,001** |
|  |  | Matauvia | -1,33 | 0,60 | 0,345 |
|  |  | Talai | 1,32 | 0,11 | **<0,001** |
|  |  | Votua | 1,78 | 0,13 | **<0,001** |
|  | Vagabia | Matauvia | 4,56 | 0,60 | **<0,001** |
|  |  | Talai | 7,20 | 0,11 | **<0,001** |
|  |  | Votua | 7,67 | 0,13 | **<0,001** |
|  | Matauvia | Talai | 2,65 | 0,60 | **<0,001** |
|  |  | Votua | 3,11 | 0,60 | **<0,001** |
|  | Talai | Votua | 0,46 | 0,12 | **<0,001** |
| 200 Hz | Nasau | Navalowara | -0,77 | 0,10 | **<0,001** |
|  |  | Savesi | -0,69 | 0,11 | **<0,001** |
|  |  | Takewa | 1,24 | 0,10 | **<0,001** |
|  |  | Vagabia | -5,39 | 0,10 | **<0,001** |
|  |  | Matauvia | -0,04 | 0,51 | 1,000 |
|  |  | Talai | 3,52 | 0,10 | **<0,001** |
|  |  | Votua | 3,93 | 0,11 | **<0,001** |
|  | Navalowara | Savesi | 0,08 | 0,10 | 0,994 |
|  |  | Takewa | 2,01 | 0,10 | **<0,001** |
|  |  | Vagabia | -4,62 | 0,10 | **<0,001** |
|  |  | Matauvia | 0,73 | 0,51 | 0,836 |
|  |  | Talai | 4,30 | 0,09 | **<0,001** |
|  |  | Votua | 4,70 | 0,11 | **<0,001** |
|  | Savesi | Takewa | 1,93 | 0,10 | **<0,001** |
|  |  | Vagabia | -4,70 | 0,10 | **<0,001** |
|  |  | Matauvia | 0,65 | 0,51 | 0,904 |
|  |  | Talai | 4,22 | 0,10 | **<0,001** |
|  |  | Votua | 4,62 | 0,11 | **<0,001** |
|  | Takewa | Vagabia | -6,63 | 0,09 | **<0,001** |
|  |  | Matauvia | -1,28 | 0,51 | 0,184 |
|  |  | Talai | 2,29 | 0,09 | **<0,001** |
|  |  | Votua | 2,69 | 0,11 | **<0,001** |
|  | Vagabia | Matauvia | 5,35 | 0,51 | **<0,001** |
|  |  | Talai | 8,92 | 0,09 | **<0,001** |
|  |  | Votua | 9,32 | 0,11 | **<0,001** |
|  | Matauvia | Talai | 3,57 | 0,51 | **<0,001** |
|  |  | Votua | 3,97 | 0,51 | **<0,001** |
|  | Talai | Votua | 0,40 | 0,10 | **<0,001** |
| 250 Hz | Nasau | Navalowara | 0,12 | 0,09 | 0,896 |
|  |  | Savesi | -2,18 | 0,10 | **<0,001** |
|  |  | Takewa | 1,30 | 0,09 | **<0,001** |
|  |  | Vagabia | -7,01 | 0,09 | **<0,001** |
|  |  | Matauvia | 0,81 | 0,45 | 0,621 |
|  |  | Talai | 4,11 | 0,09 | **<0,001** |
|  |  | Votua | 5,23 | 0,10 | **<0,001** |
|  | Navalowara | Savesi | -2,30 | 0,09 | **<0,001** |
|  |  | Takewa | 1,18 | 0,08 | **<0,001** |
|  |  | Vagabia | -7,13 | 0,09 | **<0,001** |
|  |  | Matauvia | 0,69 | 0,45 | 0,789 |
|  |  | Talai | 3,98 | 0,08 | **<0,001** |
|  |  | Votua | 5,11 | 0,10 | **<0,001** |
|  | Savesi | Takewa | 3,48 | 0,09 | **<0,001** |
|  |  | Vagabia | -4,83 | 0,09 | **<0,001** |
|  |  | Matauvia | 3,00 | 0,45 | **<0,001** |
|  |  | Talai | 6,29 | 0,09 | **<0,001** |
|  |  | Votua | 7,41 | 0,10 | **<0,001** |
|  | Takewa | Vagabia | -8,31 | 0,08 | **<0,001** |
|  |  | Matauvia | -0,49 | 0,45 | 0,961 |
|  |  | Talai | 2,80 | 0,08 | **<0,001** |
|  |  | Votua | 3,93 | 0,09 | **<0,001** |
|  | Vagabia | Matauvia | 7,82 | 0,45 | **<0,001** |
|  |  | Talai | 11,12 | 0,08 | **<0,001** |
|  |  | Votua | 12,24 | 0,09 | **<0,001** |
|  | Matauvia | Talai | 3,29 | 0,45 | **<0,001** |
|  |  | Votua | 4,42 | 0,45 | **<0,001** |
|  | Talai | Votua | 1,13 | 0,09 | **<0,001** |
| 315 Hz | Nasau | Navalowara | 1,05 | 0,09 | **<0,001** |
|  |  | Savesi | -3,06 | 0,09 | **<0,001** |
|  |  | Takewa | 1,94 | 0,09 | **<0,001** |
|  |  | Vagabia | -7,60 | 0,09 | **<0,001** |
|  |  | Matauvia | 2,26 | 0,44 | **<0,001** |
|  |  | Talai | 4,56 | 0,09 | **<0,001** |
|  |  | Votua | 5,80 | 0,10 | **<0,001** |
|  | Navalowara | Savesi | -4,11 | 0,09 | **<0,001** |
|  |  | Takewa | 0,89 | 0,08 | **<0,001** |
|  |  | Vagabia | -8,65 | 0,08 | **<0,001** |
|  |  | Matauvia | 1,21 | 0,44 | 0,109 |
|  |  | Talai | 3,51 | 0,08 | **<0,001** |
|  |  | Votua | 4,74 | 0,09 | **<0,001** |
|  | Savesi | Takewa | 4,99 | 0,09 | **<0,001** |
|  |  | Vagabia | -4,54 | 0,09 | **<0,001** |
|  |  | Matauvia | 5,32 | 0,44 | **<0,001** |
|  |  | Talai | 7,62 | 0,09 | **<0,001** |
|  |  | Votua | 8,85 | 0,10 | **<0,001** |
|  | Takewa | Vagabia | -9,53 | 0,08 | **<0,001** |
|  |  | Matauvia | 0,32 | 0,44 | 0,996 |
|  |  | Talai | 2,63 | 0,08 | **<0,001** |
|  |  | Votua | 3,86 | 0,09 | **<0,001** |
|  | Vagabia | Matauvia | 9,86 | 0,44 | **<0,001** |
|  |  | Talai | 12,16 | 0,08 | **<0,001** |
|  |  | Votua | 13,39 | 0,09 | **<0,001** |
|  | Matauvia | Talai | 2,30 | 0,44 | **<0,001** |
|  |  | Votua | 3,54 | 0,44 | **<0,001** |
|  | Talai | Votua | 1,23 | 0,09 | **<0,001** |
| 400 Hz | Nasau | Navalowara | 0,90 | 0,09 | **<0,001** |
|  |  | Savesi | -3,05 | 0,10 | **<0,001** |
|  |  | Takewa | 2,60 | 0,09 | **<0,001** |
|  |  | Vagabia | -7,20 | 0,09 | **<0,001** |
|  |  | Matauvia | 3,08 | 0,46 | **<0,001** |
|  |  | Talai | 4,39 | 0,09 | **<0,001** |
|  |  | Votua | 5,68 | 0,10 | **<0,001** |
|  | Navalowara | Savesi | -3,96 | 0,09 | **<0,001** |
|  |  | Takewa | 1,70 | 0,09 | **<0,001** |
|  |  | Vagabia | -8,10 | 0,09 | **<0,001** |
|  |  | Matauvia | 2,18 | 0,45 | **<0,001** |
|  |  | Talai | 3,49 | 0,08 | **<0,001** |
|  |  | Votua | 4,78 | 0,10 | **<0,001** |
|  | Savesi | Takewa | 5,66 | 0,09 | **<0,001** |
|  |  | Vagabia | -4,15 | 0,09 | **<0,001** |
|  |  | Matauvia | 6,13 | 0,46 | **<0,001** |
|  |  | Talai | 7,44 | 0,09 | **<0,001** |
|  |  | Votua | 8,73 | 0,10 | **<0,001** |
|  | Takewa | Vagabia | -9,80 | 0,08 | **<0,001** |
|  |  | Matauvia | 0,48 | 0,45 | 0,966 |
|  |  | Talai | 1,79 | 0,08 | **<0,001** |
|  |  | Votua | 3,08 | 0,09 | **<0,001** |
|  | Vagabia | Matauvia | 10,28 | 0,45 | **<0,001** |
|  |  | Talai | 11,59 | 0,08 | **<0,001** |
|  |  | Votua | 12,88 | 0,10 | **<0,001** |
|  | Matauvia | Talai | 1,31 | 0,45 | 0,076 |
|  |  | Votua | 2,60 | 0,46 | **<0,001** |
|  | Talai | Votua | 1,29 | 0,09 | **<0,001** |
| 500 Hz | Nasau | Navalowara | -0,20 | 0,11 | 0,559 |
|  |  | Savesi | -1,83 | 0,11 | **<0,001** |
|  |  | Takewa | 3,48 | 0,10 | **<0,001** |
|  |  | Vagabia | -5,76 | 0,10 | **<0,001** |
|  |  | Matauvia | 4,13 | 0,51 | **<0,001** |
|  |  | Talai | 3,93 | 0,10 | **<0,001** |
|  |  | Votua | 5,53 | 0,12 | **<0,001** |
|  | Navalowara | Savesi | -1,63 | 0,10 | **<0,001** |
|  |  | Takewa | 3,68 | 0,10 | **<0,001** |
|  |  | Vagabia | -5,56 | 0,10 | **<0,001** |
|  |  | Matauvia | 4,33 | 0,51 | **<0,001** |
|  |  | Talai | 4,13 | 0,10 | **<0,001** |
|  |  | Votua | 5,73 | 0,11 | **<0,001** |
|  | Savesi | Takewa | 5,31 | 0,10 | **<0,001** |
|  |  | Vagabia | -3,92 | 0,10 | **<0,001** |
|  |  | Matauvia | 5,97 | 0,51 | **<0,001** |
|  |  | Talai | 5,77 | 0,10 | **<0,001** |
|  |  | Votua | 7,36 | 0,11 | **<0,001** |
|  | Takewa | Vagabia | -9,24 | 0,10 | **<0,001** |
|  |  | Matauvia | 0,65 | 0,51 | 0,908 |
|  |  | Talai | 0,46 | 0,09 | **<0,001** |
|  |  | Votua | 2,05 | 0,11 | **<0,001** |
|  | Vagabia | Matauvia | 9,89 | 0,51 | **<0,001** |
|  |  | Talai | 9,69 | 0,09 | **<0,001** |
|  |  | Votua | 11,28 | 0,11 | **<0,001** |
|  | Matauvia | Talai | -0,20 | 0,51 | 1,000 |
|  |  | Votua | 1,39 | 0,51 | 0,120 |
|  | Talai | Votua | 1,59 | 0,11 | **<0,001** |
| 630 Hz | Nasau | Navalowara | 0,04 | 0,11 | 1,000 |
|  |  | Savesi | 0,19 | 0,11 | 0,687 |
|  |  | Takewa | 4,23 | 0,11 | **<0,001** |
|  |  | Vagabia | -2,99 | 0,11 | **<0,001** |
|  |  | Matauvia | 6,19 | 0,53 | **<0,001** |
|  |  | Talai | 5,01 | 0,11 | **<0,001** |
|  |  | Votua | 6,14 | 0,12 | **<0,001** |
|  | Navalowara | Savesi | 0,15 | 0,10 | 0,847 |
|  |  | Takewa | 4,19 | 0,10 | **<0,001** |
|  |  | Vagabia | -3,03 | 0,10 | **<0,001** |
|  |  | Matauvia | 6,15 | 0,53 | **<0,001** |
|  |  | Talai | 4,97 | 0,10 | **<0,001** |
|  |  | Votua | 6,10 | 0,11 | **<0,001** |
|  | Savesi | Takewa | 4,04 | 0,10 | **<0,001** |
|  |  | Vagabia | -3,18 | 0,10 | **<0,001** |
|  |  | Matauvia | 6,00 | 0,53 | **<0,001** |
|  |  | Talai | 4,82 | 0,10 | **<0,001** |
|  |  | Votua | 5,95 | 0,11 | **<0,001** |
|  | Takewa | Vagabia | -7,22 | 0,10 | **<0,001** |
|  |  | Matauvia | 1,96 | 0,53 | **<0,001** |
|  |  | Talai | 0,77 | 0,10 | **<0,001** |
|  |  | Votua | 1,90 | 0,11 | **<0,001** |
|  | Vagabia | Matauvia | 9,18 | 0,53 | **<0,001** |
|  |  | Talai | 8,00 | 0,10 | **<0,001** |
|  |  | Votua | 9,13 | 0,11 | **<0,001** |
|  | Matauvia | Talai | -1,18 | 0,53 | 0,319 |
|  |  | Votua | -0,06 | 0,53 | 1,000 |
|  | Talai | Votua | 1,13 | 0,11 | **<0,001** |
| 800 Hz | Nasau | Navalowara | 1,78 | 0,10 | **<0,001** |
|  |  | Savesi | 2,55 | 0,10 | **<0,001** |
|  |  | Takewa | 4,88 | 0,10 | **<0,001** |
|  |  | Vagabia | -0,57 | 0,10 | **<0,001** |
|  |  | Matauvia | 8,08 | 0,47 | **<0,001** |
|  |  | Talai | 7,80 | 0,09 | **<0,001** |
|  |  | Votua | 8,35 | 0,11 | **<0,001** |
|  | Navalowara | Savesi | 0,77 | 0,09 | **<0,001** |
|  |  | Takewa | 3,10 | 0,09 | **<0,001** |
|  |  | Vagabia | -2,35 | 0,09 | **<0,001** |
|  |  | Matauvia | 6,30 | 0,47 | **<0,001** |
|  |  | Talai | 6,02 | 0,09 | **<0,001** |
|  |  | Votua | 6,57 | 0,10 | **<0,001** |
|  | Savesi | Takewa | 2,33 | 0,09 | **<0,001** |
|  |  | Vagabia | -3,11 | 0,09 | **<0,001** |
|  |  | Matauvia | 5,54 | 0,47 | **<0,001** |
|  |  | Talai | 5,25 | 0,09 | **<0,001** |
|  |  | Votua | 5,81 | 0,10 | **<0,001** |
|  | Takewa | Vagabia | -5,45 | 0,09 | **<0,001** |
|  |  | Matauvia | 3,20 | 0,47 | **<0,001** |
|  |  | Talai | 2,92 | 0,09 | **<0,001** |
|  |  | Votua | 3,47 | 0,10 | **<0,001** |
|  | Vagabia | Matauvia | 8,65 | 0,47 | **<0,001** |
|  |  | Talai | 8,37 | 0,09 | **<0,001** |
|  |  | Votua | 8,92 | 0,10 | **<0,001** |
|  | Matauvia | Talai | -0,28 | 0,47 | 0,999 |
|  |  | Votua | 0,27 | 0,47 | 0,999 |
|  | Talai | Votua | 0,55 | 0,10 | **<0,001** |
| 1000 Hz | Nasau | Navalowara | 2,91 | 0,09 | **<0,001** |
|  |  | Savesi | 3,73 | 0,10 | **<0,001** |
|  |  | Takewa | 6,59 | 0,09 | **<0,001** |
|  |  | Vagabia | 0,50 | 0,09 | **<0,001** |
|  |  | Matauvia | 10,08 | 0,45 | **<0,001** |
|  |  | Talai | 9,80 | 0,09 | **<0,001** |
|  |  | Votua | 9,49 | 0,10 | **<0,001** |
|  | Navalowara | Savesi | 0,82 | 0,09 | **<0,001** |
|  |  | Takewa | 3,69 | 0,09 | **<0,001** |
|  |  | Vagabia | -2,41 | 0,09 | **<0,001** |
|  |  | Matauvia | 7,17 | 0,45 | **<0,001** |
|  |  | Talai | 6,89 | 0,08 | **<0,001** |
|  |  | Votua | 6,58 | 0,10 | **<0,001** |
|  | Savesi | Takewa | 2,86 | 0,09 | **<0,001** |
|  |  | Vagabia | -3,23 | 0,09 | **<0,001** |
|  |  | Matauvia | 6,34 | 0,45 | **<0,001** |
|  |  | Talai | 6,07 | 0,09 | **<0,001** |
|  |  | Votua | 5,76 | 0,10 | **<0,001** |
|  | Takewa | Vagabia | -6,10 | 0,08 | **<0,001** |
|  |  | Matauvia | 3,48 | 0,45 | **<0,001** |
|  |  | Talai | 3,20 | 0,08 | **<0,001** |
|  |  | Votua | 2,90 | 0,09 | **<0,001** |
|  | Vagabia | Matauvia | 9,58 | 0,45 | **<0,001** |
|  |  | Talai | 9,30 | 0,08 | **<0,001** |
|  |  | Votua | 8,99 | 0,09 | **<0,001** |
|  | Matauvia | Talai | -0,28 | 0,45 | 0,999 |
|  |  | Votua | -0,59 | 0,45 | 0,904 |
|  | Talai | Votua | -0,31 | 0,09 | **<0,001** |
| 1250 Hz | Nasau | Navalowara | 3,53 | 0,08 | **<0,001** |
|  |  | Savesi | 3,47 | 0,08 | **<0,001** |
|  |  | Takewa | 8,74 | 0,08 | **<0,001** |
|  |  | Vagabia | 1,90 | 0,08 | **<0,001** |
|  |  | Matauvia | 10,79 | 0,38 | **<0,001** |
|  |  | Talai | 11,39 | 0,08 | **<0,001** |
|  |  | Votua | 10,32 | 0,08 | **<0,001** |
|  | Navalowara | Savesi | -0,06 | 0,08 | 0,993 |
|  |  | Takewa | 5,21 | 0,07 | **<0,001** |
|  |  | Vagabia | -1,63 | 0,07 | **<0,001** |
|  |  | Matauvia | 7,26 | 0,38 | **<0,001** |
|  |  | Talai | 7,86 | 0,07 | **<0,001** |
|  |  | Votua | 6,79 | 0,08 | **<0,001** |
|  | Savesi | Takewa | 5,28 | 0,07 | **<0,001** |
|  |  | Vagabia | -1,57 | 0,07 | **<0,001** |
|  |  | Matauvia | 7,32 | 0,38 | **<0,001** |
|  |  | Talai | 7,93 | 0,07 | **<0,001** |
|  |  | Votua | 6,85 | 0,08 | **<0,001** |
|  | Takewa | Vagabia | -6,85 | 0,07 | **<0,001** |
|  |  | Matauvia | 2,04 | 0,38 | **<0,001** |
|  |  | Talai | 2,65 | 0,07 | **<0,001** |
|  |  | Votua | 1,58 | 0,08 | **<0,001** |
|  | Vagabia | Matauvia | 8,89 | 0,38 | **<0,001** |
|  |  | Talai | 9,50 | 0,07 | **<0,001** |
|  |  | Votua | 8,43 | 0,08 | **<0,001** |
|  | Matauvia | Talai | 0,61 | 0,38 | 0,743 |
|  |  | Votua | -0,46 | 0,38 | 0,926 |
|  | Talai | Votua | -1,07 | 0,08 | **<0,001** |
| 1600 Hz | Nasau | Navalowara | 4,51 | 0,07 | **<0,001** |
|  |  | Savesi | 3,20 | 0,07 | **<0,001** |
|  |  | Takewa | 9,81 | 0,07 | **<0,001** |
|  |  | Vagabia | 2,96 | 0,07 | **<0,001** |
|  |  | Matauvia | 11,08 | 0,33 | **<0,001** |
|  |  | Talai | 12,74 | 0,07 | **<0,001** |
|  |  | Votua | 11,14 | 0,07 | **<0,001** |
|  | Navalowara | Savesi | -1,31 | 0,07 | **<0,001** |
|  |  | Takewa | 5,30 | 0,06 | **<0,001** |
|  |  | Vagabia | -1,55 | 0,06 | **<0,001** |
|  |  | Matauvia | 6,57 | 0,33 | **<0,001** |
|  |  | Talai | 8,23 | 0,06 | **<0,001** |
|  |  | Votua | 6,63 | 0,07 | **<0,001** |
|  | Savesi | Takewa | 6,61 | 0,06 | **<0,001** |
|  |  | Vagabia | -0,24 | 0,07 | **<0,001** |
|  |  | Matauvia | 7,89 | 0,33 | **<0,001** |
|  |  | Talai | 9,54 | 0,06 | **<0,001** |
|  |  | Votua | 7,94 | 0,07 | **<0,001** |
|  | Takewa | Vagabia | -6,85 | 0,06 | **<0,001** |
|  |  | Matauvia | 1,28 | 0,33 | **<0,001** |
|  |  | Talai | 2,93 | 0,06 | **<0,001** |
|  |  | Votua | 1,33 | 0,07 | **<0,001** |
|  | Vagabia | Matauvia | 8,12 | 0,33 | **<0,001** |
|  |  | Talai | 9,77 | 0,06 | **<0,001** |
|  |  | Votua | 8,17 | 0,07 | **<0,001** |
|  | Matauvia | Talai | 1,65 | 0,33 | **<0,001** |
|  |  | Votua | 0,05 | 0,33 | 1,000 |
|  | Talai | Votua | -1,60 | 0,07 | **<0,001** |
| 2000 Hz | Nasau | Navalowara | 5,33 | 0,07 | **<0,001** |
|  |  | Savesi | 3,45 | 0,07 | **<0,001** |
|  |  | Takewa | 10,05 | 0,07 | **<0,001** |
|  |  | Vagabia | 3,64 | 0,07 | **<0,001** |
|  |  | Matauvia | 12,30 | 0,33 | **<0,001** |
|  |  | Talai | 13,59 | 0,07 | **<0,001** |
|  |  | Votua | 11,50 | 0,07 | **<0,001** |
|  | Navalowara | Savesi | -1,87 | 0,06 | **<0,001** |
|  |  | Takewa | 4,72 | 0,06 | **<0,001** |
|  |  | Vagabia | -1,69 | 0,06 | **<0,001** |
|  |  | Matauvia | 6,97 | 0,33 | **<0,001** |
|  |  | Talai | 8,27 | 0,06 | **<0,001** |
|  |  | Votua | 6,18 | 0,07 | **<0,001** |
|  | Savesi | Takewa | 6,60 | 0,06 | **<0,001** |
|  |  | Vagabia | 0,19 | 0,06 | 0,067 |
|  |  | Matauvia | 8,85 | 0,33 | **<0,001** |
|  |  | Talai | 10,14 | 0,06 | **<0,001** |
|  |  | Votua | 8,05 | 0,07 | **<0,001** |
|  | Takewa | Vagabia | -6,41 | 0,06 | **<0,001** |
|  |  | Matauvia | 2,25 | 0,33 | **<0,001** |
|  |  | Talai | 3,54 | 0,06 | **<0,001** |
|  |  | Votua | 1,46 | 0,07 | **<0,001** |
|  | Vagabia | Matauvia | 8,66 | 0,33 | **<0,001** |
|  |  | Talai | 9,95 | 0,06 | **<0,001** |
|  |  | Votua | 7,87 | 0,07 | **<0,001** |
|  | Matauvia | Talai | 1,29 | 0,33 | **<0,001** |
|  |  | Votua | -0,80 | 0,33 | 0,225 |
|  | Talai | Votua | -2,09 | 0,07 | **<0,001** |
| 2500 Hz | Nasau | Navalowara | 5,86 | 0,07 | **<0,001** |
|  |  | Savesi | 3,44 | 0,07 | **<0,001** |
|  |  | Takewa | 9,35 | 0,07 | **<0,001** |
|  |  | Vagabia | 4,46 | 0,07 | **<0,001** |
|  |  | Matauvia | 12,85 | 0,33 | **<0,001** |
|  |  | Talai | 14,10 | 0,07 | **<0,001** |
|  |  | Votua | 11,76 | 0,07 | **<0,001** |
|  | Navalowara | Savesi | -2,42 | 0,06 | **<0,001** |
|  |  | Takewa | 3,50 | 0,06 | **<0,001** |
|  |  | Vagabia | -1,40 | 0,06 | **<0,001** |
|  |  | Matauvia | 6,99 | 0,33 | **<0,001** |
|  |  | Talai | 8,24 | 0,06 | **<0,001** |
|  |  | Votua | 5,90 | 0,07 | **<0,001** |
|  | Savesi | Takewa | 5,92 | 0,06 | **<0,001** |
|  |  | Vagabia | 1,02 | 0,06 | **<0,001** |
|  |  | Matauvia | 9,41 | 0,33 | **<0,001** |
|  |  | Talai | 10,66 | 0,06 | **<0,001** |
|  |  | Votua | 8,32 | 0,07 | **<0,001** |
|  | Takewa | Vagabia | -4,90 | 0,06 | **<0,001** |
|  |  | Matauvia | 3,49 | 0,33 | **<0,001** |
|  |  | Talai | 4,74 | 0,06 | **<0,001** |
|  |  | Votua | 2,40 | 0,07 | **<0,001** |
|  | Vagabia | Matauvia | 8,39 | 0,33 | **<0,001** |
|  |  | Talai | 9,64 | 0,06 | **<0,001** |
|  |  | Votua | 7,30 | 0,07 | **<0,001** |
|  | Matauvia | Talai | 1,25 | 0,33 | **<0,001** |
|  |  | Votua | -1,09 | 0,33 | **<0,001** |
|  | Talai | Votua | -2,34 | 0,07 | **<0,001** |
| 3150 Hz | Nasau | Navalowara | 5,60 | 0,07 | **<0,001** |
|  |  | Savesi | 2,14 | 0,07 | **<0,001** |
|  |  | Takewa | 8,68 | 0,07 | **<0,001** |
|  |  | Vagabia | 5,11 | 0,07 | **<0,001** |
|  |  | Matauvia | 13,42 | 0,35 | **<0,001** |
|  |  | Talai | 13,64 | 0,07 | **<0,001** |
|  |  | Votua | 12,05 | 0,08 | **<0,001** |
|  | Navalowara | Savesi | -3,46 | 0,07 | **<0,001** |
|  |  | Takewa | 3,07 | 0,06 | **<0,001** |
|  |  | Vagabia | -0,50 | 0,07 | **<0,001** |
|  |  | Matauvia | 7,82 | 0,34 | **<0,001** |
|  |  | Talai | 8,04 | 0,06 | **<0,001** |
|  |  | Votua | 6,45 | 0,07 | **<0,001** |
|  | Savesi | Takewa | 6,54 | 0,07 | **<0,001** |
|  |  | Vagabia | 2,97 | 0,07 | **<0,001** |
|  |  | Matauvia | 11,28 | 0,35 | **<0,001** |
|  |  | Talai | 11,50 | 0,07 | **<0,001** |
|  |  | Votua | 9,91 | 0,08 | **<0,001** |
|  | Takewa | Vagabia | -3,57 | 0,06 | **<0,001** |
|  |  | Matauvia | 4,74 | 0,34 | **<0,001** |
|  |  | Talai | 4,96 | 0,06 | **<0,001** |
|  |  | Votua | 3,37 | 0,07 | **<0,001** |
|  | Vagabia | Matauvia | 8,31 | 0,34 | **<0,001** |
|  |  | Talai | 8,54 | 0,06 | **<0,001** |
|  |  | Votua | 6,95 | 0,07 | **<0,001** |
|  | Matauvia | Talai | 0,22 | 0,34 | 0,998 |
|  |  | Votua | -1,37 | 0,35 | **<0,001** |
|  | Talai | Votua | -1,59 | 0,07 | **<0,001** |
| 4000 Hz | Nasau | Navalowara | 4,98 | 0,07 | **<0,001** |
|  |  | Savesi | 1,33 | 0,08 | **<0,001** |
|  |  | Takewa | 10,43 | 0,07 | **<0,001** |
|  |  | Vagabia | 4,46 | 0,07 | **<0,001** |
|  |  | Matauvia | 13,69 | 0,36 | **<0,001** |
|  |  | Talai | 13,24 | 0,07 | **<0,001** |
|  |  | Votua | 12,02 | 0,08 | **<0,001** |
|  | Navalowara | Savesi | -3,65 | 0,07 | **<0,001** |
|  |  | Takewa | 5,45 | 0,07 | **<0,001** |
|  |  | Vagabia | -0,52 | 0,07 | **<0,001** |
|  |  | Matauvia | 8,71 | 0,36 | **<0,001** |
|  |  | Talai | 8,26 | 0,07 | **<0,001** |
|  |  | Votua | 7,04 | 0,08 | **<0,001** |
|  | Savesi | Takewa | 9,09 | 0,07 | **<0,001** |
|  |  | Vagabia | 3,13 | 0,07 | **<0,001** |
|  |  | Matauvia | 12,35 | 0,36 | **<0,001** |
|  |  | Talai | 11,91 | 0,07 | **<0,001** |
|  |  | Votua | 10,69 | 0,08 | **<0,001** |
|  | Takewa | Vagabia | -5,97 | 0,07 | **<0,001** |
|  |  | Matauvia | 3,26 | 0,36 | **<0,001** |
|  |  | Talai | 2,81 | 0,06 | **<0,001** |
|  |  | Votua | 1,60 | 0,07 | **<0,001** |
|  | Vagabia | Matauvia | 9,23 | 0,36 | **<0,001** |
|  |  | Talai | 8,78 | 0,07 | **<0,001** |
|  |  | Votua | 7,56 | 0,07 | **<0,001** |
|  | Matauvia | Talai | -0,45 | 0,36 | 0,915 |
|  |  | Votua | -1,66 | 0,36 | **<0,001** |
|  | Talai | Votua | -1,22 | 0,07 | **<0,001** |
| 5000 Hz | Nasau | Navalowara | 5,30 | 0,07 | **<0,001** |
|  |  | Savesi | 1,18 | 0,08 | **<0,001** |
|  |  | Takewa | 8,07 | 0,07 | **<0,001** |
|  |  | Vagabia | 3,02 | 0,07 | **<0,001** |
|  |  | Matauvia | 10,28 | 0,36 | **<0,001** |
|  |  | Talai | 13,16 | 0,07 | **<0,001** |
|  |  | Votua | 10,24 | 0,08 | **<0,001** |
|  | Navalowara | Savesi | -4,12 | 0,07 | **<0,001** |
|  |  | Takewa | 2,76 | 0,07 | **<0,001** |
|  |  | Vagabia | -2,28 | 0,07 | **<0,001** |
|  |  | Matauvia | 4,98 | 0,36 | **<0,001** |
|  |  | Talai | 7,86 | 0,07 | **<0,001** |
|  |  | Votua | 4,94 | 0,08 | **<0,001** |
|  | Savesi | Takewa | 6,88 | 0,07 | **<0,001** |
|  |  | Vagabia | 1,84 | 0,07 | **<0,001** |
|  |  | Matauvia | 9,10 | 0,36 | **<0,001** |
|  |  | Talai | 11,97 | 0,07 | **<0,001** |
|  |  | Votua | 9,05 | 0,08 | **<0,001** |
|  | Takewa | Vagabia | -5,05 | 0,07 | **<0,001** |
|  |  | Matauvia | 2,22 | 0,36 | **<0,001** |
|  |  | Talai | 5,09 | 0,07 | **<0,001** |
|  |  | Votua | 2,17 | 0,07 | **<0,001** |
|  | Vagabia | Matauvia | 7,26 | 0,36 | **<0,001** |
|  |  | Talai | 10,14 | 0,07 | **<0,001** |
|  |  | Votua | 7,22 | 0,07 | **<0,001** |
|  | Matauvia | Talai | 2,87 | 0,36 | **<0,001** |
|  |  | Votua | -0,05 | 0,36 | 1,000 |
|  | Talai | Votua | -2,92 | 0,07 | **<0,001** |
| 6300 Hz | Nasau | Navalowara | 3,63 | 0,07 | **<0,001** |
|  |  | Savesi | -0,95 | 0,08 | **<0,001** |
|  |  | Takewa | 7,52 | 0,07 | **<0,001** |
|  |  | Vagabia | 2,65 | 0,07 | **<0,001** |
|  |  | Matauvia | 9,63 | 0,36 | **<0,001** |
|  |  | Talai | 13,18 | 0,07 | **<0,001** |
|  |  | Votua | 9,66 | 0,08 | **<0,001** |
|  | Navalowara | Savesi | -4,58 | 0,07 | **<0,001** |
|  |  | Takewa | 3,89 | 0,07 | **<0,001** |
|  |  | Vagabia | -0,98 | 0,07 | **<0,001** |
|  |  | Matauvia | 6,00 | 0,36 | **<0,001** |
|  |  | Talai | 9,55 | 0,07 | **<0,001** |
|  |  | Votua | 6,03 | 0,08 | **<0,001** |
|  | Savesi | Takewa | 8,47 | 0,07 | **<0,001** |
|  |  | Vagabia | 3,60 | 0,07 | **<0,001** |
|  |  | Matauvia | 10,58 | 0,36 | **<0,001** |
|  |  | Talai | 14,13 | 0,07 | **<0,001** |
|  |  | Votua | 10,61 | 0,08 | **<0,001** |
|  | Takewa | Vagabia | -4,86 | 0,07 | **<0,001** |
|  |  | Matauvia | 2,11 | 0,35 | **<0,001** |
|  |  | Talai | 5,66 | 0,06 | **<0,001** |
|  |  | Votua | 2,14 | 0,07 | **<0,001** |
|  | Vagabia | Matauvia | 6,98 | 0,36 | **<0,001** |
|  |  | Talai | 10,53 | 0,07 | **<0,001** |
|  |  | Votua | 7,01 | 0,07 | **<0,001** |
|  | Matauvia | Talai | 3,55 | 0,35 | **<0,001** |
|  |  | Votua | 0,03 | 0,36 | 1,000 |
|  | Talai | Votua | -3,52 | 0,07 | **<0,001** |
| 8000 Hz | Nasau | Navalowara | 3,53 | 0,07 | **<0,001** |
|  |  | Savesi | 1,58 | 0,07 | **<0,001** |
|  |  | Takewa | 6,82 | 0,07 | **<0,001** |
|  |  | Vagabia | 3,00 | 0,07 | **<0,001** |
|  |  | Matauvia | 9,80 | 0,35 | **<0,001** |
|  |  | Talai | 12,78 | 0,07 | **<0,001** |
|  |  | Votua | 9,46 | 0,08 | **<0,001** |
|  | Navalowara | Savesi | -1,96 | 0,07 | **<0,001** |
|  |  | Takewa | 3,29 | 0,07 | **<0,001** |
|  |  | Vagabia | -0,54 | 0,07 | **<0,001** |
|  |  | Matauvia | 6,26 | 0,35 | **<0,001** |
|  |  | Talai | 9,25 | 0,07 | **<0,001** |
|  |  | Votua | 5,93 | 0,07 | **<0,001** |
|  | Savesi | Takewa | 5,24 | 0,07 | **<0,001** |
|  |  | Vagabia | 1,42 | 0,07 | **<0,001** |
|  |  | Matauvia | 8,22 | 0,35 | **<0,001** |
|  |  | Talai | 11,20 | 0,07 | **<0,001** |
|  |  | Votua | 7,88 | 0,08 | **<0,001** |
|  | Takewa | Vagabia | -3,83 | 0,07 | **<0,001** |
|  |  | Matauvia | 2,97 | 0,35 | **<0,001** |
|  |  | Talai | 5,96 | 0,06 | **<0,001** |
|  |  | Votua | 2,64 | 0,07 | **<0,001** |
|  | Vagabia | Matauvia | 6,80 | 0,35 | **<0,001** |
|  |  | Talai | 9,78 | 0,06 | **<0,001** |
|  |  | Votua | 6,47 | 0,07 | **<0,001** |
|  | Matauvia | Talai | 2,98 | 0,35 | **<0,001** |
|  |  | Votua | -0,33 | 0,35 | 0,981 |
|  | Talai | Votua | -3,32 | 0,07 | **<0,001** |
| 10000 Hz | Nasau | Navalowara | 3,04 | 0,07 | **<0,001** |
|  |  | Savesi | 2,18 | 0,07 | **<0,001** |
|  |  | Takewa | 6,37 | 0,07 | **<0,001** |
|  |  | Vagabia | 2,81 | 0,07 | **<0,001** |
|  |  | Matauvia | 9,87 | 0,34 | **<0,001** |
|  |  | Talai | 12,86 | 0,07 | **<0,001** |
|  |  | Votua | 9,32 | 0,08 | **<0,001** |
|  | Navalowara | Savesi | -0,86 | 0,07 | **<0,001** |
|  |  | Takewa | 3,33 | 0,06 | **<0,001** |
|  |  | Vagabia | -0,24 | 0,06 | **<0,001** |
|  |  | Matauvia | 6,83 | 0,34 | **<0,001** |
|  |  | Talai | 9,82 | 0,06 | **<0,001** |
|  |  | Votua | 6,28 | 0,07 | **<0,001** |
|  | Savesi | Takewa | 4,19 | 0,07 | **<0,001** |
|  |  | Vagabia | 0,62 | 0,07 | **<0,001** |
|  |  | Matauvia | 7,69 | 0,34 | **<0,001** |
|  |  | Talai | 10,67 | 0,07 | **<0,001** |
|  |  | Votua | 7,14 | 0,07 | **<0,001** |
|  | Takewa | Vagabia | -3,56 | 0,06 | **<0,001** |
|  |  | Matauvia | 3,50 | 0,34 | **<0,001** |
|  |  | Talai | 6,49 | 0,06 | **<0,001** |
|  |  | Votua | 2,95 | 0,07 | **<0,001** |
|  | Vagabia | Matauvia | 7,06 | 0,34 | **<0,001** |
|  |  | Talai | 10,05 | 0,06 | **<0,001** |
|  |  | Votua | 6,51 | 0,07 | **<0,001** |
|  | Matauvia | Talai | 2,99 | 0,34 | **<0,001** |
|  |  | Votua | -0,55 | 0,34 | 0,745 |
|  | Talai | Votua | -3,54 | 0,07 | **<0,001** |
| 12500 Hz | Nasau | Navalowara | 3,01 | 0,07 | **<0,001** |
|  |  | Savesi | 3,79 | 0,07 | **<0,001** |
|  |  | Takewa | 5,71 | 0,07 | **<0,001** |
|  |  | Vagabia | 2,71 | 0,07 | **<0,001** |
|  |  | Matauvia | 9,88 | 0,33 | **<0,001** |
|  |  | Talai | 12,91 | 0,07 | **<0,001** |
|  |  | Votua | 9,24 | 0,07 | **<0,001** |
|  | Navalowara | Savesi | 0,79 | 0,06 | **<0,001** |
|  |  | Takewa | 2,71 | 0,06 | **<0,001** |
|  |  | Vagabia | -0,30 | 0,06 | **<0,001** |
|  |  | Matauvia | 6,87 | 0,33 | **<0,001** |
|  |  | Talai | 9,90 | 0,06 | **<0,001** |
|  |  | Votua | 6,23 | 0,07 | **<0,001** |
|  | Savesi | Takewa | 1,92 | 0,06 | **<0,001** |
|  |  | Vagabia | -1,08 | 0,06 | **<0,001** |
|  |  | Matauvia | 6,08 | 0,33 | **<0,001** |
|  |  | Talai | 9,12 | 0,06 | **<0,001** |
|  |  | Votua | 5,44 | 0,07 | **<0,001** |
|  | Takewa | Vagabia | -3,00 | 0,06 | **<0,001** |
|  |  | Matauvia | 4,16 | 0,33 | **<0,001** |
|  |  | Talai | 7,20 | 0,06 | **<0,001** |
|  |  | Votua | 3,52 | 0,07 | **<0,001** |
|  | Vagabia | Matauvia | 7,16 | 0,33 | **<0,001** |
|  |  | Talai | 10,20 | 0,06 | **<0,001** |
|  |  | Votua | 6,53 | 0,07 | **<0,001** |
|  | Matauvia | Talai | 3,03 | 0,33 | **<0,001** |
|  |  | Votua | -0,64 | 0,33 | 0,514 |
|  | Talai | Votua | -3,67 | 0,07 | **<0,001** |
| 16000 Hz | Nasau | Navalowara | 3,25 | 0,07 | **<0,001** |
|  |  | Savesi | 3,62 | 0,07 | **<0,001** |
|  |  | Takewa | 6,82 | 0,06 | **<0,001** |
|  |  | Vagabia | 2,10 | 0,07 | **<0,001** |
|  |  | Matauvia | 10,49 | 0,32 | **<0,001** |
|  |  | Talai | 14,05 | 0,06 | **<0,001** |
|  |  | Votua | 9,26 | 0,07 | **<0,001** |
|  | Navalowara | Savesi | 0,37 | 0,06 | **<0,001** |
|  |  | Takewa | 3,57 | 0,06 | **<0,001** |
|  |  | Vagabia | -1,15 | 0,06 | **<0,001** |
|  |  | Matauvia | 7,24 | 0,32 | **<0,001** |
|  |  | Talai | 10,79 | 0,06 | **<0,001** |
|  |  | Votua | 6,00 | 0,07 | **<0,001** |
|  | Savesi | Takewa | 3,20 | 0,06 | **<0,001** |
|  |  | Vagabia | -1,52 | 0,06 | **<0,001** |
|  |  | Matauvia | 6,87 | 0,32 | **<0,001** |
|  |  | Talai | 10,44 | 0,06 | **<0,001** |
|  |  | Votua | 5,64 | 0,07 | **<0,001** |
|  | Takewa | Vagabia | -4,72 | 0,06 | **<0,001** |
|  |  | Matauvia | 3,67 | 0,32 | **<0,001** |
|  |  | Talai | 7,22 | 0,06 | **<0,001** |
|  |  | Votua | 2,44 | 0,07 | **<0,001** |
|  | Vagabia | Matauvia | 8,39 | 0,32 | **<0,001** |
|  |  | Talai | 11,94 | 0,06 | **<0,001** |
|  |  | Votua | 7,15 | 0,07 | **<0,001** |
|  | Matauvia | Talai | 3,55 | 0,32 | **<0,001** |
|  |  | Votua | -1,24 | 0,32 | **<0,001** |
|  | Talai | Votua | -4,79 | 0,07 | **<0,001** |
